# *In Silico* Molecular Comparisons of *C. elegans* and Mammalian Pharmacology Identify Distinct Targets That Regulate Feeding

**DOI:** 10.1371/journal.pbio.1001712

**Published:** 2013-11-19

**Authors:** George A. Lemieux, Michael J. Keiser, Maria F. Sassano, Christian Laggner, Fahima Mayer, Roland J. Bainton, Zena Werb, Bryan L. Roth, Brian K. Shoichet, Kaveh Ashrafi

**Affiliations:** 1 Department of Anatomy, University of California, San Francisco, California, United States of America; 2 SeaChange Pharmaceuticals Inc., San Francisco, California, United States of America; 3 Department of Pharmaceutical Chemistry, University of California, San Francisco, California, United States of America; 4 Department of Pharmacology, University of North Carolina Medical School, Chapel Hill, North Carolina, United States of America; 5 Department of Physiology, University of California, San Francisco, California, United States of America; 6 Department of Anesthesiology, University of California, San Francisco, California, United States of America; Brandeis University, United States of America

## Abstract

Phenotypic screens can identify molecules that are at once penetrant and active on the integrated circuitry of a whole cell or organism. These advantages are offset by the need to identify the targets underlying the phenotypes. Additionally, logistical considerations limit screening for certain physiological and behavioral phenotypes to organisms such as zebrafish and *C. elegans*. This further raises the challenge of elucidating whether compound-target relationships found in model organisms are preserved in humans. To address these challenges we searched for compounds that affect feeding behavior in *C. elegans* and sought to identify their molecular mechanisms of action. Here, we applied predictive chemoinformatics to small molecules previously identified in a *C. elegans* phenotypic screen likely to be enriched for feeding regulatory compounds. Based on the predictions, 16 of these compounds were tested *in vitro* against 20 mammalian targets. Of these, nine were active, with affinities ranging from 9 nM to 10 µM. Four of these nine compounds were found to alter feeding. We then verified the *in vitro* findings *in vivo* through genetic knockdowns, the use of previously characterized compounds with high affinity for the four targets, and chemical genetic epistasis, which is the effect of combined chemical and genetic perturbations on a phenotype relative to that of each perturbation in isolation. Our findings reveal four previously unrecognized pathways that regulate feeding in *C. elegans* with strong parallels in mammals. Together, our study addresses three inherent challenges in phenotypic screening: the identification of the molecular targets from a phenotypic screen, the confirmation of the *in vivo* relevance of these targets, and the evolutionary conservation and relevance of these targets to their human orthologs.

## Abstract 2

This paper takes advantage of similarities between the *C. elegans* and human pharmacopeia to identify and validate pharmacological targets that regulate *C. elegans* feeding rates.

## Author Summary

Many beneficial pharmacological interventions were first discovered by observing the effects of perturbation of intact biological systems by small organic molecules without *a priori* knowledge of their targets. This forward pharmacological approach has the advantage of directly identifying new pharmacological agents that are active on complex biological processes. However, because of experimental feasibility, systematic application of this approach is generally limited to small animals such as the roundworm *C. elegans* and zebrafish, raising the question of whether use of these animals could identify compounds that act on ortholgous mammalian targets. A significant challenge in addressing this question is the determination of the molecular identities of the compounds' targets responsible for the desired phenotypic outcomes. Here we describe a computational approach for target identification based on structural similarities of newly identified compounds to known ligand interactions with mostly mammalian targets. For several of the compounds emerging from a *C. elegans* phenotypic screen, we predict and confirm mammalian targets using *in vitro* binding assays. Using genetic and pharmacological assays, we then demonstrate that a subset of these compounds alter *C. elegans* feeding rates through the *C. elegans* counterparts of the predicted mammalian targets.

## Introduction

Before the molecular biology era, pharmacological targets were typically classified by the effects of organic molecules on whole tissues [1]. Many pathways were first recognized based on phenotypic responsiveness to compounds without knowledge of underlying molecular mechanisms. Examples include the inference of the α- and β-adrenergic pathways in the 1940s [2], the inference of the H2 histaminergic receptor [3] and of the μ, and κ-opioid receptors in the 1970s [4], and the proposal of the 5-HT3 serotonergic receptor in the mid-1980s [5]. Although these targets were eventually characterized by molecular biology, the tissue and organism approach had the advantage that the compounds emerging from it were active on a physiologically intact tissue or organismal circuit, and directly linked functional perturbation of targets to biological effects.

Phenotypic compound screens return to this classical approach to capture some of the same advantages for the discovery of molecules with systemic activity. Such screens have generally relied on high content microscopy assays in cell-based systems [6]–[8]. However, certain biological processes such as physiology and behavior are the result of integrated organism-wide processes that only manifest themselves in intact multicellular organisms. For example, as a physiological process, feeding behavior is the outcome of integration of extrinsic and intrinsic cues of food availability and energy demand and thus is best understood when studied in whole organisms. Elucidation of the neural circuits that determine feeding is a fundamental challenge in the neuroscience of energy homeostasis [9]. Small molecules that alter feeding behavior can serve as useful reagents for investigating these circuits and provide exquisite temporal control in ways not easily achieved through genetic manipulations.

Given their small size and ease of manipulation, *C. elegans* have been used in pharmacology-based phenotypic screens [10]–[14]. These animals are also well suited for study of molecular and neural circuits that underlie food intake behavior. *C. elegans* feeds using peristaltic contractions of a muscular pharynx to aspirate microbes into the lumen of the intestine [15]. This pharyngeal pumping rate directly correlates with the transport of nutrients into the intestinal lumen [16],[17]. *C. elegans*' central nervous system integrates signals from external cues such as food availability, food quality, and internal nutritional status to regulate feeding behavior [17]–[20]. Multiple pathways in the nervous system that are dependent on serotonin, glutamate, and neuropeptide release regulate the pharyngeal pumping rate in *C. elegans* [21]–[27]. Thus, *C. elegans*' feeding behavior is subject to regulation by some of the same physiological parameters and molecular components as those in mammals.

Feeding behavior is a relatively challenging read-out for a screen-scale phenotypic effort. Therefore, we focused on surrogate phenotypes that could potentially enrich for identification of feeding regulatory compounds. From a high-content microscopy assay, we previously discovered 84 compounds, which increased or decreased Nile Red in *C. elegans* [11]. Nile Red is a vital dye that has been broadly used for detecting fat levels in numerous experimental systems [28]–[32]. However, its use as a read-out of fat content in *C. elegans* has been challenged [33]–[36]. Nevertheless, for a subset of compounds emerging from the initial Nile Red screen, effects on lipid content were further verified by other vital dyes, biochemical methods, molecular read-outs of fat content, as well as efficacy in mammalian cell-based models of adipogenesis [11]. Therefore, we hypothesized that compounds emerging from the Nile Red screen would be enriched for those that also alter feeding behavior.

For all but one of the compounds, no biological targets were previously known. To predict targets for these compounds, we used chemoinformatic inference based on ligand patterns against mammalian receptors. For a subset of the predictions, we tested the compounds against the predicted mammalian targets *in vitro* and subsequently tested orthologs of these targets in *C. elegans* by chemical-genetic epistasis. We then combined the compounds with the mutants to map epistasis relationships for feeding behavior, identifying four signaling pathways previously unassociated with *C. elegans* feeding regulation. Together, our findings reveal that chemical screens in *C. elegans* lead to molecules with activity toward known human targets and highlight the utility of *C. elegans* for unambiguous assessment of compound–target–phenotype relationships in the context of an intact organism.

## Results

### *C. elegans* Screen Actives Are Similar to Ligands for Multiple Mammalian Targets

Computational chemoinformatics methods have been used to query annotated ligand–target interactions to identify novel targets for known drugs [37] and, recently, targets for ligands identified by a screen in zebrafish [38]. We used the Similarity Ensemble Approach (SEA) [39] to interrogate the ChEMBL database for targets, represented by their ligand sets, resembling compounds of the 84 identified in a *C. elegans* phenotypic screen [11]. ChEMBL annotates more than 8,000,000 ligand–target interactions for 2,456 targets; mostly, mammalian. SEA uses extended connectivity fingerprints to measure chemical similarity, quantified as Tanimoto coefficients (T*_c_*), between a query molecule and each target ligand. The ensemble of all pairwise T*_c_'s* for a target's ligand set to the query are summed and compared to an expectation of random association. Using statistical machinery similar to BLAST, an expectation value (*E*-value) quantifies the possibility that observed structural similarities could occur by chance. Because SEA relies on reported ligand–target interactions, it cannot predict associations for chemically novel molecules or for targets for which no ligands have been identified. Nevertheless, SEA provides a rapid and systematic approach for discovering the pharmacological relevance of *C. elegans* actives vis-à-vis mammalian targets with known ligands.

**Figure 1:**
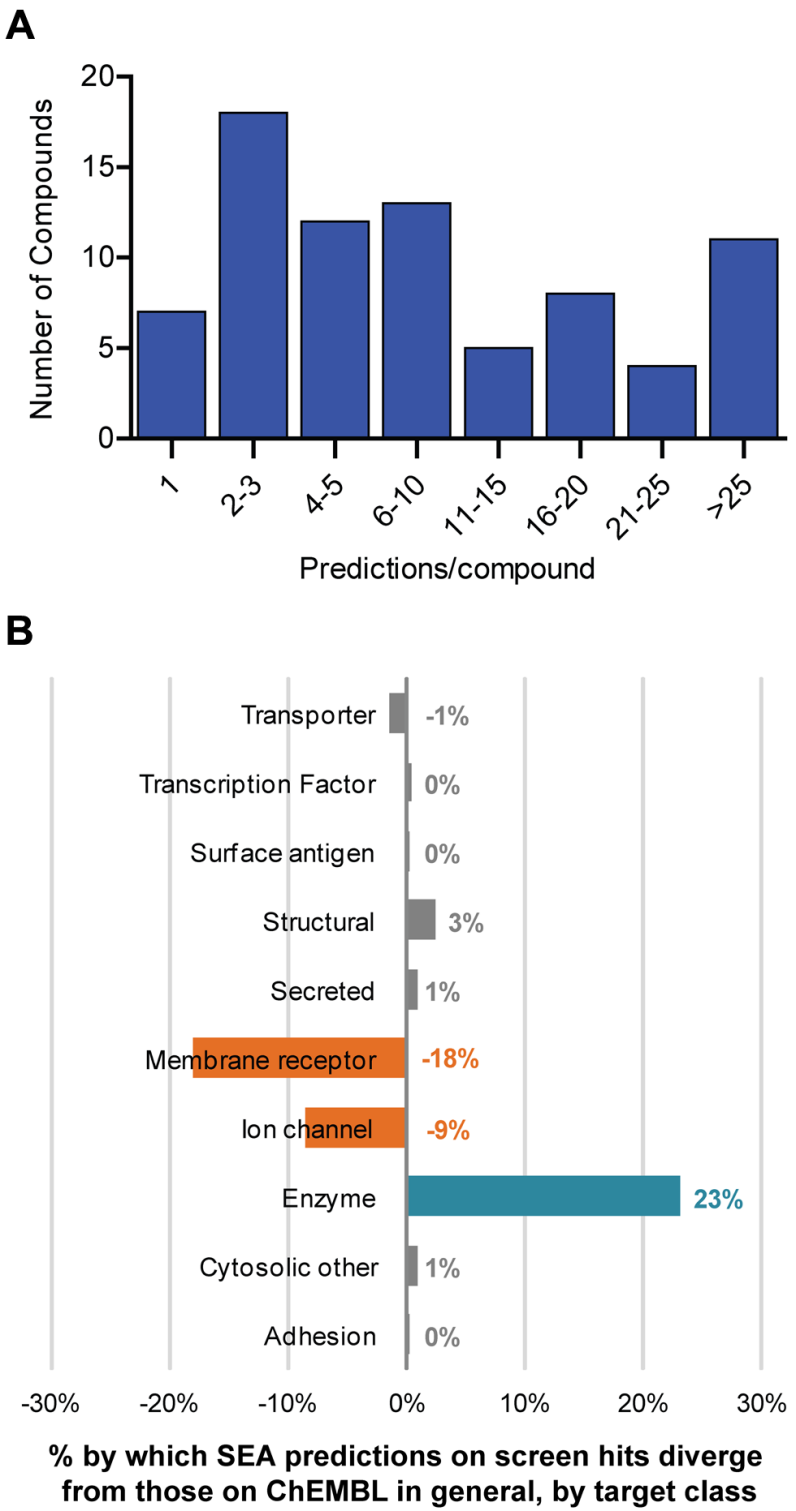
Overview of the ligand-target predictions for *C. elegans* screen actives. (A) Distribution of ligand predictions per compound expressed as a histogram. (B) Target classes more frequently (positive %) or less frequently (negative %) predicted for *C. elegans* screen actives, using predictions on ChEMBL's ligands as a baseline. Data are calculated based on ligand–target interactions at a minimum significance threshold of *E*<0.00001.

At an *E*-value threshold of less than 10^−5^, 79 of the 84 active compounds were associated with at least one target in ChEMBL. Most compounds were predicted to have two or more targets, with 572 distinct targets predicted overall (Figure 1A). Target predictions often spanned two or more classes per compound as has been observed in GPCR pharmacological relationships [40]. We first considered the possibility that the profile of predicted targets simply recapitulates the underlying distribution of target classes in ChEMBL. This was not the case. Our ChEMBL-derived dataset is composed of 35% enzymes, 15% ion channels, 30% membrane receptors, and 20% transporters, transcription factors, and other proteins. Conversely, more than 60% of the SEA-predicted targets for the phenotypic actives were enzymes, whereas both membrane receptors and ion channels were comparatively underrepresented (Figure 1B). This deviation may reflect the targets with known ligands that are relevant to metabolic phenotypes or peculiarities of the *C. elegans* model, a question that we will address below.

### Testing SEA Predictions of Mammalian Targets *in Vitro*

**Figure 2:**
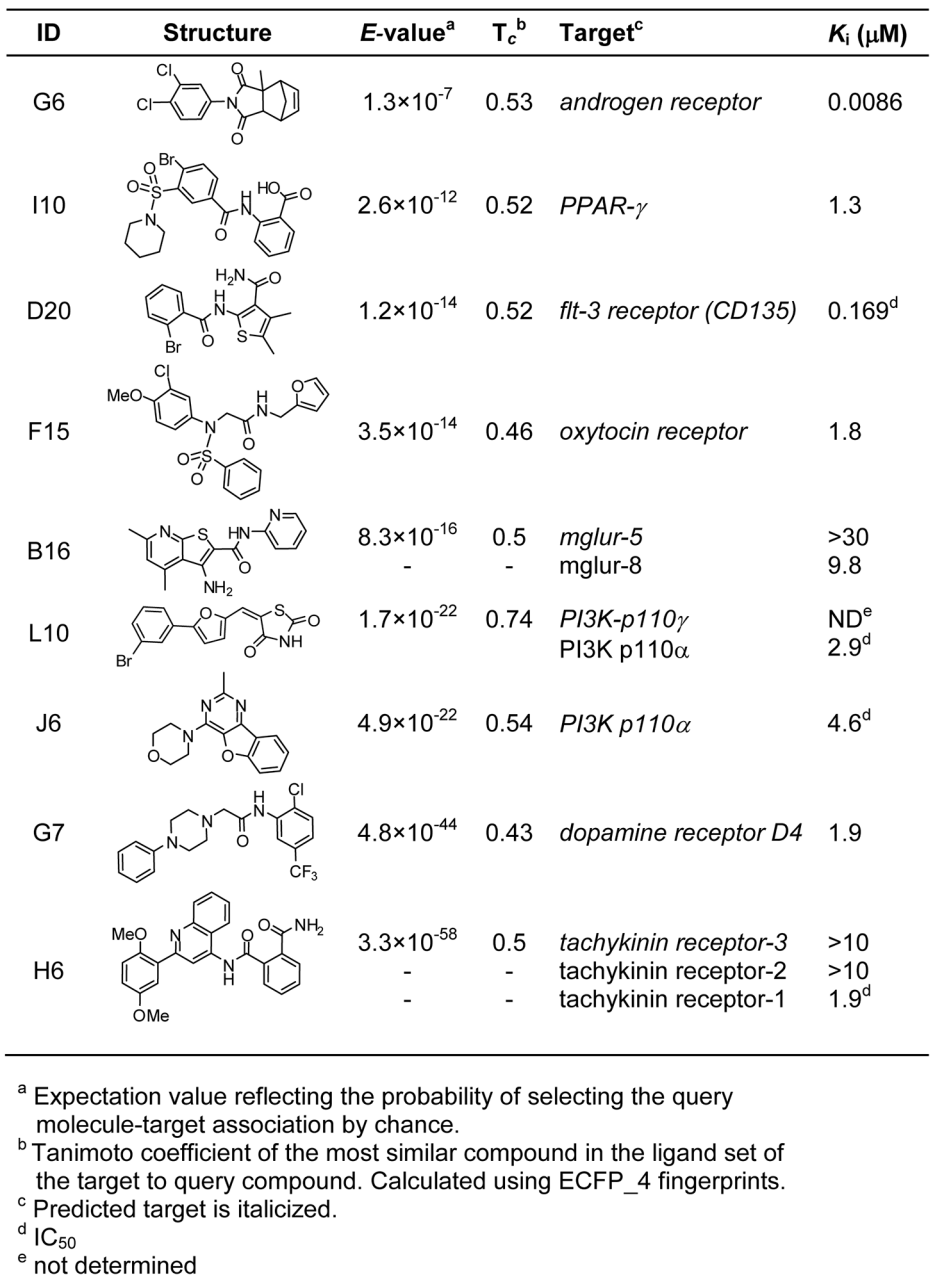
SEA predictions that were confirmed by *in vitro* testing.

To test the SEA predictions, we assayed selected molecules *in vitro* against their putative mammalian targets. Given the many potential ligand–target interactions (79 ligands on 572 targets, 1,024 overall pairs), we only tested compound–target interactions for which established assays were readily accessible. We tested 16 different compounds against 20 targets (21 ligand–target pairs) ranging from G-protein coupled receptors (GPCRs) and nuclear hormone receptors (NHRs) to kinases and phosphatases (Figure 2, Table S1). Nine compounds had significant activity at a 10 µM assay concentration against nine predicted targets, including the dopamine, tachykinin, oxytocin, and metabotropic glutamate receptors, the Flt-3 receptor tyrosine kinase, PI3-kinases, and the NHRs peroxisome proliferator activator receptor-gamma (PPAR-γ) and the androgen receptor (Figure 2). Significant activity was not observed for 12 target–ligand predictions *in vitro* (Table S1). A final ligand–target prediction did not show activity on the human receptor *in vitro*, but was confirmed by chemical-genetic epistasis in *C. elegans* (below). This 43% *in vitro* hit rate resembles that observed for chemoinformatic linkage of human drugs to new [37] and to adverse drug reaction targets [41].

The potencies of the hits ranged from 8.6 nM to 9.8 µM in full concentration response analysis (Figure 2, Figures S1, S2, S3). As expected, there was no correlation between *E*-value and potency, since target affinity is not considered when calculating ligand similarities. In several cases, SEA successfully predicted the overall target family but not the exact isoform. For instance, H6 and B16 were predicted to antagonize tachykinin receptor–3 (Tkr-3) and metabotropic glutamate receptor 5 (mGluR-5), respectively, but instead antagonized Tkr-1 and mGluR-8 (Figure 2). Indeed, compared to other mGluR antagonists, B16 appears to be uniquely specific for the mGluR8 isoform (Table S2).

### Identification of *C. elegans* Feeding-Regulatory Compounds

**Figure 3:**
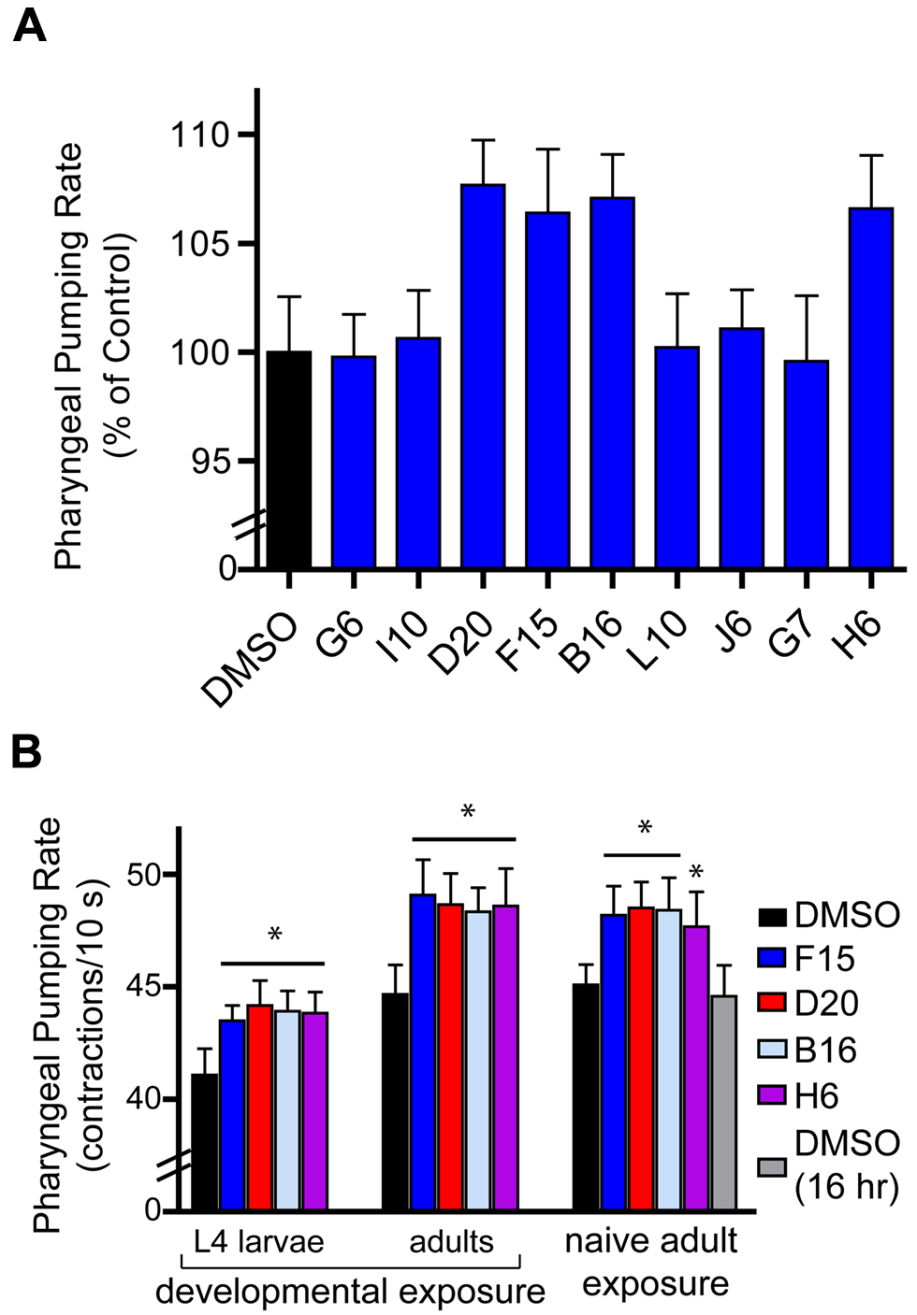
Several compounds with predicted and confirmed human targets increase pharyngeal pumping. (A) Wild-type *C. elegans* were cultured on media supplemented with either 0.1% DMSO (vehicle control) or 10 µM of each compound. (B) The effects of the compounds on the pharyngeal pumping rate when exposed for differing developmental periods was evaluated for *C. elegans* exposed to each 10 uM of each compound during different times: L1 to L4 (2 d at 20°C), L1 to gravid adult (3 d at 20°C, and naïve day 1 gravid adults exposed to B16, F15, and D20 for 1 h, H6 for 16 h). The pharyngeal pumping rate of 10–13 animals per condition was quantified. Error bars represent the standard deviation. *p<0.01: ANOVA, Dunnett's multiple comparisons test. In (B) gravid adults exposed to H6 for 16 h was compared to DMSO 16 h (*t* test: two tailed *p<0.01).

To search for feeding regulatory compounds whose SEA-predicted targets could be confirmed by direct testing against mammalian targets *in vitro*, we focused on the compounds listed in Figure 2. We found that B16, H6, F15, and D20 each increased the pharyngeal pumping rate (Figure 3A). Our standard assay for pharyngeal pumping rate is a real-time assay over a short time interval (10 s), in which contractions of the posterior bulb of the pharynx are manually counted. The rapid assay is particularly amenable to measuring pumping rate in large numbers of animals and experimental conditions. However, to alleviate concerns that the short duration of the assay may capture an unrepresentative aspect of the pumping rate, we compared its results with those obtained over a longer time course (60 s) using time-lapse microscopy (Figure S4, Movies S1, S2, S3, S4, S5). The long time course study revealed that for young, egg-laying gravid adults foraging on *E. coli* OP-50 lawns, the *C. elegans* pharynx contracts almost continuously with occasional brief pauses of less than a second, similar to that seen during short-term measurements. Importantly, the results of the long-term video measurements confirmed our short-term manual protocol that B16, H6, F15, and D20 all increase pharyngeal pumping in a range of 7%–12%. The percent increase in pharyngeal pumping rate caused by these compound treatments is within the physiological range *C. elegans* exhibits in response to food following a fast, or that reported with animals treated with serotonin [17],[27], one of the best characterized modulators of *C. elegans* pharyngeal pumping. While reduced pharyngeal pumping could reflect deleterious effects on animal health such as a general disruption of neuromuscular junctions, increased pumping is less likely to be due to such nonspecific effects.

To determine whether the feeding increasing effects of B16, H6, F15, and D20 were dependent on specific developmental stages, we evaluated their effects when administered at different developmental exposure times (Figure 3B). *C. elegans* treated with compounds beginning at the first larval stage (L1) assayed as L4 animals exhibited similar percentage increases in feeding in response to B16, H6, F15, and D20 as animals that were allowed an extra day under treatment then assayed as adults. L4 animals do have a basal pumping rate that is lower than adults, and this is reflected in different absolute pump counts for the two stages (Figure 3B). Developmental exposure to the compounds was not required to elevate the pumping rate, since day 1 gravid adults raised without compound exposure still exhibited elevated pumping once exposed to these compounds. The feeding elevating effects of B16, D20, and F15 were notable within 1 h of exposure but that of H6 required ∼16 h (Figure 3B).

### Pharmacological Similarity Predictions Identify Feeding Regulatory Genes in *C. elegans*

To ascertain whether the feeding increasing compounds mediated their effects through the targets identified in Figure 2, we used genetic epistasis to test target engagement *in vivo*. We first examined compound D20, which was predicted and shown *in vitro* to act on the human Flt-3 receptor tyrosine kinase, a member of the PDGF-β receptor superfamily that is involved in the early stages of hematopoiesis and is active in certain cancers [42]. While *C. elegans* do not have hematopoiesis, their genome encodes dozens of tyrosine kinase domains with sequence similarity to the human Flt-3 receptor with no one sequence being an obvious candidate (Table S3). To determine whether D20 interacted with any of these kinase domains to regulate feeding, we measured the pharyngeal pumping rates of RNAi-treated populations, treated with either D20 or DMSO as a vehicle control. We reasoned that if D20 exerts its effects through a receptor tyrosine kinase in *C. elegans*, inactivation of such a receptor should mimic the effects of D20 on feeding and, importantly, render pharyngeal pumping insensitive to further modulation. In contrast, combined pharmacological and genetic perturbations that act through independent pathways will exhibit additive or synergistic effects on feeding when combined.

**Figure 4:**
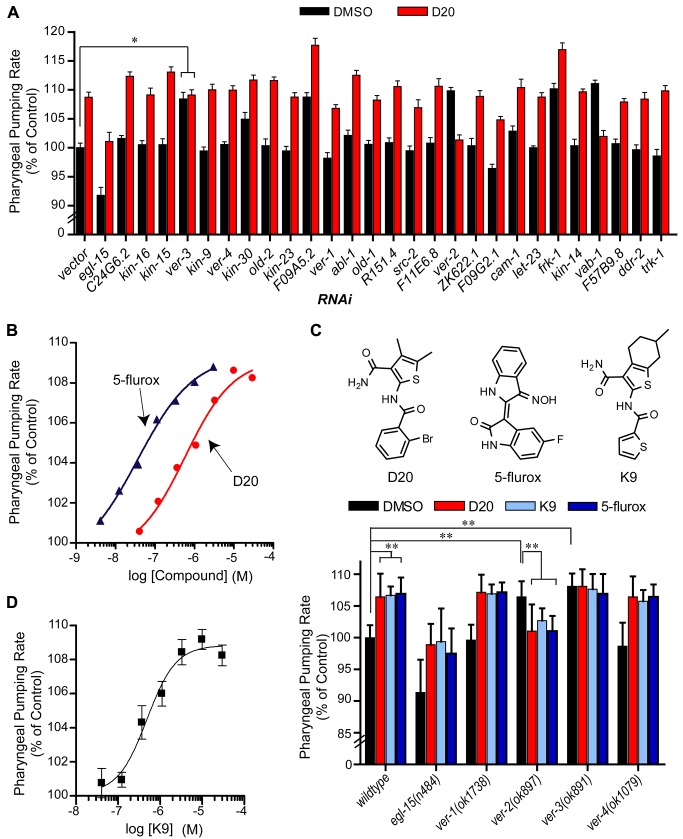
D20 antagonizes a VER-3–dependent pathway to induce pharyngeal pumping. (A) Animals were cultured on different RNAi clones in media supplemented with either 0.05% DMSO or 5 µM D20. Sequences are arranged based on their BLASTp similarity to the human FLT-3 receptor from most similar (left) to least similar (right). Error bars represent the s.e.m. **p<0.001, *p<0.01 (D20 versus DMSO): two-tailed *t* test. (B) Pharyngeal pumping rates of wild-type *C. elegans* treated with serial 3-fold dilutions of 5-flurox or D20. (C) The pharyngeal pumping rates of wild-type, *egl-15*(*n484*), *ver-1*(*ok1738*), *ver-2*(*ok897*), *ver-3*(*ok891*), and *ver-4*(*ok1079*) mutant animals cultured on 0.1% DMSO, 10 µM D20, 10 µM K9, or 2 µM 5-flurox. Error bars represent the standard deviation. Significance levels: **p<0.001 were determined by one-way ANOVA using Bonferroni's multiple comparison test. (D) *C. elegans* were treated with serial 3-fold dilutions of K9 and the pharyngeal pumping rate was quantified at each dose. Error bars represent the s.e.m. In (A–D) 10–20 animals were evaluated per condition.

For each of 26 receptor tyrosine kinases with significant BLAST similarity to the Flt-3 receptor, we examined the effects of their gene knockdowns on pharyngeal pumping with or without D20 treatment (Figure 4A). Among the 26 receptor tyrosine kinases, RNAi exposure of only one resulted in an interaction insensitive to D20 treatment—that of the VEGF-related receptor encoded by *ver-3* (Figure 4A). *C. elegans* subjected to *ver-3* RNAi exhibit an elevated pharyngeal pumping phenotype relative to vector control-treated animals, thus mimicking the effects of D20 treatment (Figure 4A). While some of the other kinase knockdowns elevated the feeding rate, they all remained sensitive to further modulation by D20. For example, while *F09A5.2* RNAi and *frk-1* RNAi each caused increased pumping relative to RNAi vector control, D20 treatment further increased pumping rates of these RNAi-treated animals (Figure 4A). Thus, the resistance of *ver-3* mutants to further enhancement of feeding by D20 was not simply due to an upper physiological limit on the pumping rate. Because pharyngeal pumping is regulated by the nervous system and RNAi is sometimes ineffective at gene knockdown, particularly in the *C. elegans* nervous system [43], it is possible that some of the intended gene products could not be sufficiently knocked down by our RNAi strategy. However, using RNAi to knock down gene expression products of each of *ver-3*, *ver-2*, *egl-15*, *and vab-1*, all of which have reported nervous system expressions (Table S3), led to effects on pharyngeal pumping that were similar to those obtained when we examined mutants in each of these genes (Figure 4C). Thus, the combination of *in vitro* binding assays and patterns of phenotypic interactions *in vivo* strongly supported the notion that D20 mediates its feeding increasing effects in a *ver-3*–dependent mechanism.

To further compare the pharmacological parallels between Flt-3 inhibitors and pharyngeal pumping, we tested a known Flt-3 receptor inhibitor (5′-fluoroindirubinoxime: 5-flurox, Figure 4B) for its effects on pharyngeal pumping. D20 induces a dose-dependent increase in the pharyngeal pumping rate with an EC_50_ of 600 nM (Figure 4B). Similarly, 5-flurox mimicked the dose-dependent effect of D20 with an EC_50_ of 40 nM (Figure 4B). The increased *in vivo* efficacy correlates with the measured *in vitro* activities of the Flt-3 receptor for D20 (*K*_i_ = 165 nM) and 5-flurox (IC_50_ = 15 nM) [44]. Finally, joint D20 and 5-flurox treatment did not increase pharyngeal pumping rate beyond that seen by individual compound treatments (Figure S5A). These combined results suggest that a target pharmacologically similar to the Flt-3 receptor exists in *C. elegans* to regulate pharyngeal pumping.

To verify the RNAi results and further ascertain that D20 and 5-flurox elicit similar effects on *C. elegans* feeding behavior, we focused on *ver-3* and its closest family members, *ver-1*, *ver-2*, *ver-4*, and *egl-15*. Similar to the RNAi results, mutants in either *ver-2* or *ver-3* exhibited elevated rates of pharyngeal pumping, while those of *ver-1* and *ver-4* resembled wild-type, and *egl-15* mutants had reduced rates of pumping (Figure 4C). As with D20, the elevated feeding rates of *ver-3* mutants were insensitive to further increase with 5-flurox treatment (Figure 4C). In contrast, treatment of *egl-15*, *ver-1*, and *ver-4* receptor mutants with either D20 or 5-flurox led to similar increases in pumping rate despite different basal pumping rates of these mutants. Treatment of the *ver-2* mutants, whose basal pumping rate was increased relative to WT, returned to untreated, WT rates with either compound, recapitulating the effect observed when D20 treatment was combined with *ver-2*(*RNAi*) (Figure 4A). The reason for this antagonistic relationship is unclear to us but may reflect a dependence of D20's feeding elevated phenotype on intact *ver-2* signaling, perhaps due to a compensatory mechanism between the different receptors. These observations support the notion that the tyrosine kinase receptor VER-3 is pharmacologically orthologous to the human Flt-3 receptor and responsible for D20's feeding phenotype.

SEA predictions may prove informative for finding mechanistic targets in *C. elegans* even if they fail to modulate the predicted mammalian targets. This appears to be the case for K9, an analog of D20 also predicted to inhibit Flt-3 kinase (Table S1, Figure 4C). K9-treated *C. elegans* resembled D20-treated animals in the dose-dependent increase in feeding rate observed (Figure 4D). In addition, K9 exhibited the same genetic interactions as D20- and 5-flurox–treated animals: wild-type, *egl-15*, *ver-1*, and *ver-4* mutants all exhibited elevated pharyngeal pumping (Figure 4C); *ver-3* mutants were insensitive to treatment; and the fast pumping *ver-2* mutant reverted to the wild-type rate upon K9 treatment. Therefore, the similarity of the pharmacological response between the *C. elegans ver-3* and the human Flt-3 receptors, while substantial, is clearly not identical.

**Figure 5:**
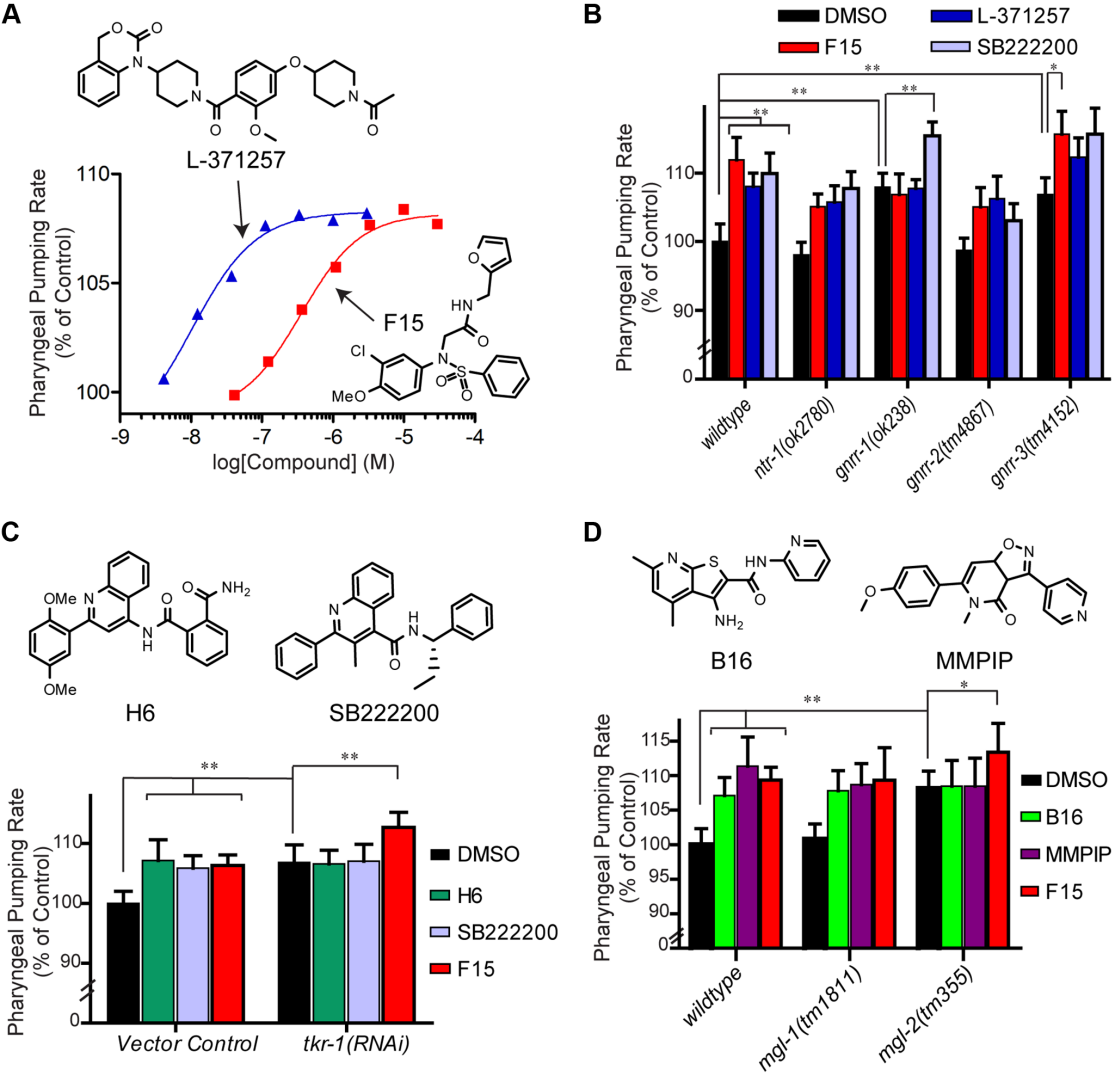
Identification of *C. elegans* GPCR-regulated feeding pathways that are pharmacologically orthologous to their human targets. (A) Wild-type *C. elegans* treated with serial 3-fold dilutions of either F15 or L-371257. Error bars represent the standard error of the mean. (B) Wild-type, *ntr-1*(*ok2780*), *gnrr-1*(*ok238*), *gnrr-2*(*tm4867*), or *gnrr-3*(*tm4152*) mutant animals cultured on either 0.1% DMSO, 10 µM F15, 100 nM L-371257, or 200 nM SB222200. (C) Wild-type *C. elegans* cultured on *E. coli* expressing either *tkr-1 RNAi* or vector control, then treated with either 0.1% DMSO,10 µM H6, 10 µM F15, or 200 nM SB222200. (D) Wild-type, *mgl-1*(*tm1811*), and *mgl-2*(*tm355*) mutant *C. elegans* cultured on media containing either 0.1% DMSO, 10 µM B16, 10 µM F15, or 2 µM MMPIP. Error bars represent the standard deviation. (A–D) The mean pharyngeal pumping rate of 10–20 *C. elegans* per condition are shown. Significance levels: **p<0.001, *p<0.05 were determined by one-way ANOVA using Bonferroni's multiple comparison test.

Based on the SEA and *in vitro* binding data, we next examined the possibility that an oxytocin receptor-like system may underlie the feeding regulatory effects of F15 (Figure 2). The oxytocin receptor system modulates mammalian feeding [45]–[47], but the existence of a *C. elegans* oxytocin receptor ortholog with a role in feeding regulation has not been defined. Both F15 and a structurally independent oxytocin receptor antagonist L-371257 exhibited dose-dependent increases in *C. elegans* pharyngeal pumping with *in vivo* EC_50_'s of 400 and 10 nM, respectively (Figure 5A). Like D20 and 5-flurox, their *in vivo C. elegans* efficacies parallel their *in vitro* affinities toward the human receptor (Oxtr *K*_i_'s for F15, 1.6 µM and for L-371257, 4.6 nM) [48]. Furthermore, simultaneous treatment with F15 and L-371257 did not further elevate pumping (Figure S5B), suggesting the existence of a target with pharmacological similarity to the human oxytocin receptor that regulates feeding.

BLAST comparisons suggest multiple *C. elegans* sequences with similarity to the human oxytocin receptor. These include an oxytocin/vasopressin-like receptor NTR-1 (T07D10.2) that modulates male mating behavior [49] and associative learning [50], a tachykinin receptor-like protein TKR-1, and three related receptors (GNRR-1, -2, -3) (Table S3). We examined the pharyngeal pumping rates in *ntr-1* and the *gnrr-1*, *-2*, and *-3* mutants (Figure 5B) and animals treated with *tkr-1* RNAi (Figure 5C). Both *ntr-1* and *gnrr-2* mutants exhibited wild-type feeding rates and were sensitive to F15- and L-371257-induced pharyngeal pumping increases. Both *gnrr-1* and *-3* mutants (Figure 5B) as well as *tkr-1*(*RNAi*) animals (Figure 5C) exhibited elevated pumping in the absence of compound treatment. However, only the pharyngeal pumping rate of the *gnrr-1* mutants was resistant to further increase by F15 and oxytocin-antagonizing L-371257 treatments (Figure 5B,C). Turning to the tachykinin system, each of wild-type, *ntr-1*, *gnrr-1*, *-2*, and *-3* mutants responded with increased feeding on treatment with SB222200, a high-affinity human tachykinin receptor antagonist (Figure 5C). In contrast to their F15 sensitivity, *tkr-1*(*RNAi*)–treated animals were indeed insensitive to the pharyngeal pumping rate increases elicited by either SB222200 or H6 treatment (Figure 5C), a compound identified in our *C. elegans* phenotypic screen and shown *in vitro* to act on a human tachykinin receptor (Figure 2). Wild-type H6-dosed animals were insensitive to SB222200-induced feeding increase (Figure S5C) consistent with their *in vitro* activities as tachykinin receptor antagonists. Together these results indicate that a tachykinin-like and an oxytocin-like receptor pathway function in parallel to regulate pharyngeal pumping in *C. elegans*. Despite relative similarities in sequence and feeding phenotype, *gnrr-1* and *tkr-1* mutants were differentiated by their antagonists and pharmacologically linked to the human oxytocin and tachykinin receptors, based on responsiveness to F15/L-371257 and H6/SB222200, respectively.

Finally we tested whether B16, the mammalian mGluR-8 inhibitor, regulates the pharyngeal pumping rate through any of the *C. elegans* metabotropic glutamate receptors. The *C. elegans* genome encodes three orthologs of human mGluR-8: *mgl-1*, *-2*, and *-3* [51]. These receptors are expressed in the *C. elegans* nervous system and regulate diverse aspects of *C. elegans* behavior and physiology, but have not previously been implicated in feeding [52]. We found that *mgl-2* but not *mgl-1* mutant animals exhibited an elevated pharyngeal pumping rate in the absence of B16 treatment (Figure 5D). The elevated feeding of *mgl-2* mutants resembled that of WT animals treated with B16, and that of animals treated with MMPIP [53], a human mGluR-7 allosteric antagonist (IC_50_'s, 26–220 nM) structurally distinct from B16 (Figure 5D). Combined treatment of wild-type animals with B16 and MMPIP resulted in no further increase over either alone (Figure S5D). The pharyngeal pumping rate of *mgl-2* mutant animals, unlike both WT and *mgl-1* mutant animals, was unaffected by treatment with either B16 or MMPIP, consistent with the notion that both agents mediate their feeding phenotype though inhibition of MGL-2. Similar to WT and *mgl-1* mutants, *mgl-2* mutant animals further elevated pumping when treated with the Oxtr/GNRR-1 antagonist F15 (Figure 5D). The additive effect of F15 on the *mgl-2* mutants' pharyngeal pumping rate distinguishes its underlying biological mechanism from that of the mGluR/*mgl-2* antagonists.

### The Regulatory Relationships of the Newly Identified Feeding Pathways

**Figure 6:**
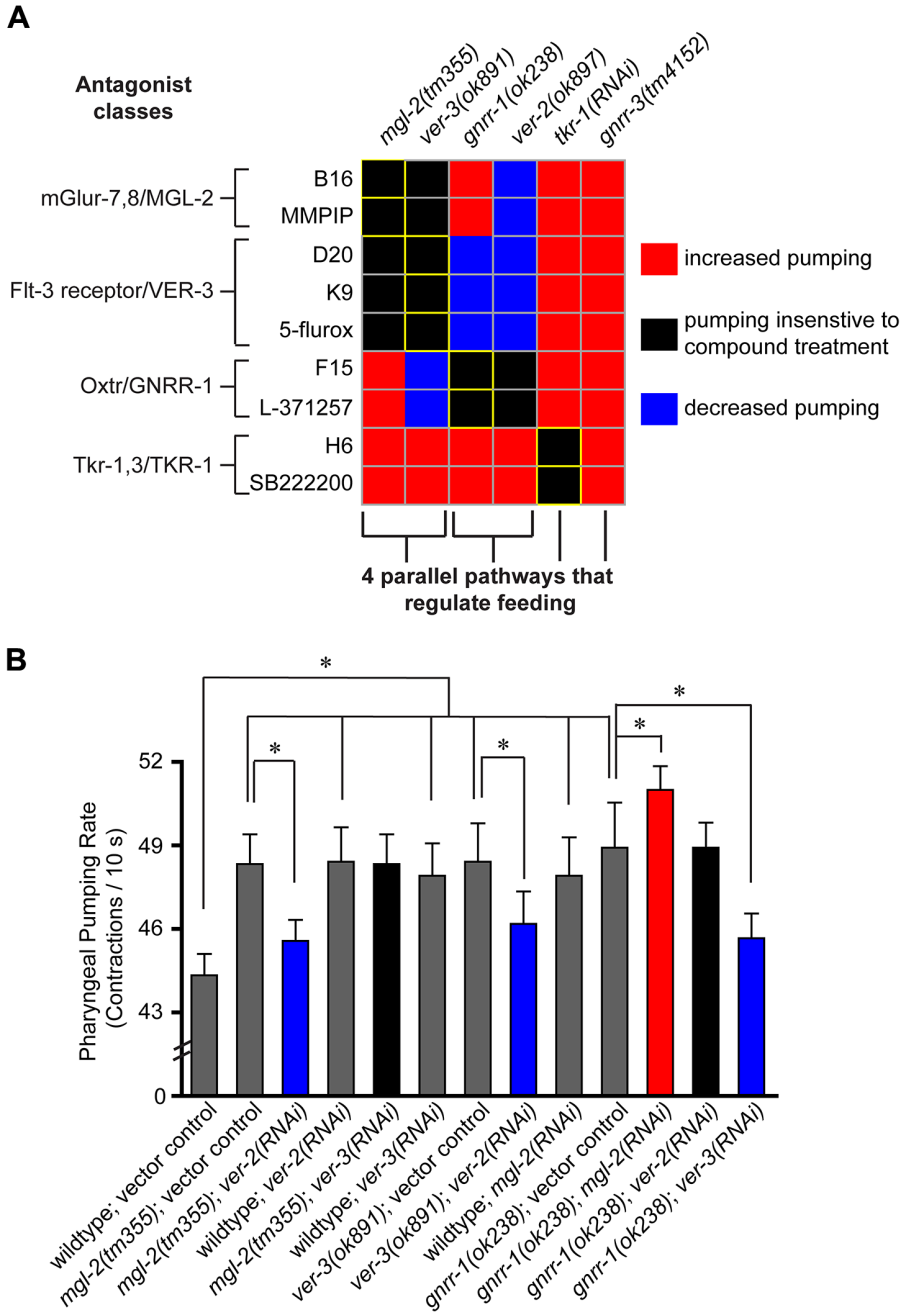
Interaction matrix of all binary combinations of compounds and gene knockdowns that individually increase pharyngeal pumping. (A) The differences in the pumping rates of compound-treated versus vehicle (0.1% DMSO) treatment on each genetic background for all pair-wise combinations of compounds and mutants were evaluated. Compound concentrations used were 10 µM for B16, D20, K9, F15, and H6; 200 nM for L-371257 and SB 222200; and 2 µM for MMPIP, 5-flurox. The predicted compound–target interactions are outlined in yellow. Red- and blue-labeled interactions indicate pumping rates significantly different (ANOVA, *p*<0.05 Dunnett's multiple comparison test) from the corresponding vehicle control-treated mutant. (B) The implied genetic interactions on pharyngeal pumping of *mgl-2*, *ver-2*, *ver-3*, and *gnrr-1* mutants assayed by mutant–RNAi combinations. Twelve animals per condition were analyzed. Error bars represent 1 standard deviation. * *p*<0.001 one-way ANOVA using Bonferroni's multiple comparison test.

To further examine the *in vivo* specificity of the compound-induced feeding phenotypes and determine whether each target regulates feeding independently of one another, we examined the feeding phenotypes of all possible binary combinations of compounds with the high pumping mutants identified by this study (Table S4). The resulting 54-interaction matrix (Figure 6A) classifies interactions between compounds and mutants based on the sensitivity of a mutant to a compound's effect on pharyngeal pumping. Considering that relative to wild-type animals on vehicle control each of the gene knockdowns and compound treatments individually were sufficient to cause feeding increases, two patterns of interactions were expected: interactions where the feeding increasing effects are additive, representing likely parallel mechanisms of actions, and those that are nonadditive, suggesting a single regulatory pathway. Pharyngeal pumping rates that exceeded those observed in the vehicle-treated mutant controls are classified as additive. For example, both H6 and SB222200 increase the pharyngeal pumping rates of *mgl-2*, *ver-3*, *gnrr-1*, *ver-2*, and *gnrr-3* mutants, a series of additive interactions, but not that of animals treated with *tkr-1* RNAi, a genetic inactivation in their common target.

We also noted 10 interactions in which elevated pumping rates of specific mutants were lowered upon compound treatment. For instance, while each of D20, K9, and 5-flurox treatments alone, as does inactivation of *ver-2*, elevate feeding rates in wild-type and in many mutant backgrounds, treatment of *ver-2* mutants with each of these compounds lowers feeding (Figure 4C, Figure 6A). Similar antagonistic patterns were also seen, for example, when *ver-3* mutants were treated with the Oxtr antagonists F15 and L-371257. While the precise reasons for antagonistic interactions are not known, one likely possibility is that distinct signaling pathways normally act in compensatory manners that are revealed by the simultaneous inhibition of both of these pathways.

Strikingly, we noted that in all cases where compounds share a common target, for example F15, L-371257, and the oxytocin receptor, both compounds exhibited identical interactions, unique to each compound pair across the mutant series. This includes additive, nonadditive, and antagonist interactions. This result strongly supports the hypothesis that the compounds share a common *in vivo* target identified by this study that drives the feeding phenotype. Beyond specificity, this matrix also indicates a higher level pathway organization of these mutants (Figure 6A). Both antagonists of the *mgl-2* pathway (B16 and MMPIP) and VER-3 inhibitors (D20, K9, and 5-flurox) interact nonadditively and reciprocally with *ver-3 and mgl-2* mutants, indicating that these gene products may act in a common pathway. However, the additive effect of B16/MMPIP versus the antagonistic phenotypic effect of D20/K9/5-flurox activity on *gnrr-1* mutants is consistent with their targets being distinct entities. In addition, GNRR-1 antagonists (F15 and L-371257) interact nonadditively with *ver-2* mutants and antagonistically with *ver-3*, recapitulating the antagonistic interaction of VER-3 inhibitors with *ver-2* mutants (Figure 4C, Figure 6A). This indicates that a second feeding regulatory pathway combines GNRR-1 and VER-2 signaling.

To evaluate whether the chemical-genetic epistasis interactions observed were the result of pharmacological peculiarities or could be confirmed by standard genetic epistasis tests, we tested genetic interactions in the implied *mgl-2/ver-3* and *ver-2/gnrr-1* pathways. The feeding rate of *ver-3* mutants was insensitive to effects of *mgl-2*(*RNAi*), but was reduced by *ver*-2(*RNAi*) (Figure 5B). *Mgl-2*(*RNAi*) interacted additively with *gnrr-1* mutants to increase the pumping rate, and *ver-3*(*RNAi*) antagonized the feeding increasing effects of the *gnrr-1* mutation (Figure 5B). *Ver*-2(*RNAi*) interacted nonadditively with the *gnrr-1* mutants and was antagonistic to *mgl-2* mutants (Figure 5B). Similar interactions between *gnrr-1* mutants and *ver-3*(*RNAi*), *ver-2*(*RNAi*), and *mgl-2*(*RNAi*) were observed by measuring pumping over longer intervals by time lapse microscopy (Figure S6). These results indicate that the chemical-genetic interactions observed in this study accurately predict the interactions of loss-of-function perturbation combinations on the pharyngeal pumping rate. As in chemical-genetic interactions, examination of mutant combinations also indicated that *mgl-2/ver-3* and *ver-2/gnrr-1* function in parallel pathways but with significant crosstalk in regulating the pharyngeal pumping rate.

## Discussion

Whole organism phenotypic screens retain key advantages of classical pharmacological approaches, such as the discovery of compounds that are biologically active and that alter physiologically intact, integrated circuits without predisposed conceptions as to which circuits should be targeted. To prove biologically informative, this forward pharmacological approach requires the determination of *in vivo* molecular targets as well as the mode of action by which the phenotype is modulated [54]. Five key observations emerge from this study. First, by chemoinformatic inference, targets may be rapidly prioritized for experimental testing on isolated receptors *in vitro*. Whereas this method did not always succeed, the confirmation of the predicted targets was high enough, at 43%, to be practical. Second, as the identified targets are overwhelmingly mammalian, the ease of phenotypic screening strategies in *C. elegans* can be linked to identification of human-relevant targets. Third, in a model system such as *C. elegans*, the relationships of orthologous targets to *in vivo* phenotypes can be parsed by applying the rationale of genetic epistasis analysis. This is critical for unambiguous *in vivo* establishment of mechanisms of action, as *in vitro* activities of even highly characterized compounds are only suggestive of the *in vivo* efficacy targets. Fourth, despite significant differences in primary sequence identity of the targets, the *in vivo* efficacy in *C. elegans* can reflect the *in vitro* activity against human targets. Finally, the chemical-genetic interactions described in this study illuminate four previously uncharacterized, parallel molecular targets that regulate food intake. Together, these findings demonstrate an experimental and computational path from phenotypic screens in *C. elegans* to the discovery of human-relevant targets and elucidation of mechanisms of actions of newly identified compounds in *C. elegans*.

Most drugs interact with multiple targets *in vivo* [41], which can confound the assignment of phenotypic effects to particular targets. This can be especially true of compounds emerging from screening campaigns prior to any efforts aimed at optimizing the potential specificities of compounds. An advantage of a pharmacological approach in *C. elegans* is that genetic perturbations can test target engagement *in vivo*. Identifying a chemical-genetic epistatic interaction does not imply that a given compound has absolute specificity for a particular target in a biological system. However, the epistasis interaction does confirm that the compound induces a particular phenotype specifically through its interaction with the pathway defined by the genetic perturbation. Thus, in a manner similar to classical double mutant analysis, chemical and genetic interactions are combined to interrogate the pharmacological relevance of hypothesized target interactions to specific phenotypes.

Our findings suggest that there is a substantial pharmacological intersection between mammals and *C. elegans*. These results indicate that a *C. elegans* phenotypic screen can lead to identification of compounds that are sufficiently similar to the mammalian pharmacopeia to allow for prediction and confirmation of their interactions with mammalian targets. In turn, evolutionary conservation of these targets, in both sequence and ligand recognition, makes it possible to accurately predict the *C. elegans* target whose perturbation results in the phenotype. It could have easily been the case that the compounds emerging from a *C. elegans* screen are so diverse as to belie prediction of targets, and that the *C. elegans* phenotypes could be irrelevant to the human target space, or could reflect new targets not previously seen. As such, it may be astonishing that this approach worked at all. In fact, 79 of 84 active compounds could be chemoinformatically linked to human targets, suggesting that even a diversity library retains substantial and, for our purposes, highly useful biases towards previously “liganded” targets. Moreover, whereas these *in vitro* mammalian targets need have no relevance for *C. elegans in vivo* pharmacology, for compounds with confirmed activity *in vitro*, orthologous targets were indeed found to mediate their *C. elegans* phenotypes.

Whether the specific compounds identified from the *C. elegans* screen act on mammalian feeding and regulatory systems remains to be determined. However, there are already some hints that functionally related circuits modulate feeding behavior in both mammals and *C. elegans*. For instance, several independent studies in chickens, mice, and rats indicate that administration of oxytocin reduces food intake [55]. Oxytocin appears to be a target of satiety signals since a lipid-related signal, oleoylethanolamide, positively requires intact hypothalamic oxytocin signaling to mediate its anorexigenic effects [45]. Conversely, hyperphagia associated with a high-fat diet requires synaptotagmin-4–mediated suppression of oxytocin vesicle exocytosis [46]. Furthermore, a key function of neurons of the hypothalamus that stimulate feeding involves the inhibition of a separate population of oxytocin neurons [47]. Thus, analogous to *C. elegans gnrr-1*, signaling through the oxytocin receptor is a negative regulator of food intake in mice. Similarly, central administration of substance P, a TKR ligand, inhibits feeding in chicks [56], consistent with the negative feeding regulatory role of the TKR-1 in *C. elegans*. While the Flt-3 receptor has no known role in the mammalian nervous system, closely related growth factor receptors, such as the platelet-derived growth factor–β (PDGF-β) receptor, are expressed in the hypothalamus, and administration of PDGF-β depresses food intake and anti-PDGF-β antibodies elevate food intake in rats [57]. This resembles our observation that loss-of-function in a *C. elegans* receptor tyrosine kinase (*ver-3*) through either mutation or pharmacological inhibition elevates pharyngeal pumping. These targets may thus have ancient evolutionary origins in the regulation of feeding behavior.

Key weaknesses of our approach merit discussion. First, whereas it is comforting that we can predict targets for most biologically active synthetic compounds even in a “diversity” library, this also reflects the restricted chemical-target space in which the field is working. A library composed of genuinely novel chemotypes might be more likely to illuminate unprecedented targets, a widely desired goal of the field. On the other hand, any such truly diverse library risks missing that small part of chemical space that is relevant to terrestrial biology, the bias towards which is, after all, a pragmatic advantage of the current libraries [58]. Second, even within this restricted ligand–target space, the chemoinformatic linkage was far from perfect, and about half of the tested compounds remain unlinked to predicted targets. While the inferential computational approach cannot replace experiment, it is a rapid, comprehensive, and quantitative assessment of biologically active small molecules with unknown mechanism. These predictions generate testable hypotheses with regards to *in vivo* function. Third, while for feeding, the *in vitro* and *in vivo* data provided a compelling case for mechanisms of actions of F15, H6, B16, and D20, it remains to be determined whether these compounds also act on as-of-yet undetermined molecular pathways to alter other biological processes in *C. elegans*. Finally, while the link between compounds, targets, and *C. elegans* phenotypes now seems strong for several of the active compounds, linkage between *C. elegans* and mammalian *in vivo* pharmacology remains to be drawn.

In summary, whereas chemoinformatic linkage retains important liabilities, its success rate here and in earlier studies [37]–[39],[41] is high enough to be pragmatic for target hypothesis testing. Similarly, despite critical differences between *C. elegans* and mammals, some of them target-based, some biology-based, the targets and ligand networks for a substantial number of small molecules are conserved enough to allow target and phenotypic association across phyla. We envision that such chemical-genetic epistasis maps could be extended to saturation mapping of the pharmacological target networks underlying feeding regulation and other processes in *C. elegans*. The amenability of *C. elegans* to genetic manipulation and pharmacological screening may find broad utility as a means to identify new small molecules with interesting phenotypes and human-relevant targets.

## Materials and Methods

### Chemicals

I10, G7, A5, and L15 were purchased from Chembridge. A15, D20, K9, G6, L10, H6, and F14 were purchased from SPECS. B16, N10, and F15 were purchased from Princeton Biomolecular Research. J16 was purchased from TimTec. MMPIP hydrochloride, SB222200, 100 nM L-371257, and 5-fluoroindirubinoxime were purchased from Tocris Biosciences. All other chemicals were purchased from Sigma.

### Computational Analysis

We computationally screened 84 phenotypically active compounds against molecular target panels from the ChEMBL database (http://www.ebi.ac.uk/chembl) using the Similarity Ensemble Approach (SEA) [37],[39] operating on 1,024-bit folded Scitegic ECFP_4 fingerprints [59] and Tanimoto coefficients as previously described [37].

For the target panel, we first used ChEMBL_7 (released November 11, 2010) and later moved to an updated 2,482 molecular target panel derived from ChEMBL_11 (released August 9, 2011). We filtered reference ligands by molecular weight (≤1,000 Da) and by reported affinity (≤10 µM), and then subjected all ligand structures to cleaning, standardization, and de-duplication as before [39].

### *In Vitro* Compound Assays against Mammalian Targets

Tachykinin, ghrelin, and calcium-sensing receptor activity were measured using a cell-based Ca flux assay by Multispan, Inc. (Hayward, California). Cholescystokinin A and B receptors were assayed for effects on cAMP production, and nicotinic acid receptor activity was measured in a cell-based assay of forskolin-stimulated cAMP production by Multispan, Inc. Calcium-sensing receptor, PP2A phosphatase activity assays were performed by CEREP (Celle l'Evescault, France) using PP2A from human erythrocytes. Phospholipase C from *Bacillus cereus* was assayed by CEREP, Inc. using glycero-phosphatidyl ethanolamine as a substrate, monitoring diacyl glycerol production. Radioligand binding assays for PPAR-α, γ, δ and the androgen receptor were performed by CEREP, Inc. CARNA Biosciences (Kobe, Japan) performed *in vitro* kinase activity assays using recombinant catalytic domains and measured phosphorylation of an Src-derived peptide for Flt-3 and phosphatidyl inositol for PI3KCA. Radioligand binding assays for the oxytocin receptor, D4 dopamine receptor, cannabinoid CB2 receptor, and angiotensin type I and II receptors were performed as described previously, as were cell-based activity assays for mGluR 1a, 2, 4, 5, 6, and 8 [60]–[62].

### *C. elegans* Strains

Strains containing *mgl-1*(*tm1811*) *X*, *mgl-2*(*tm355*) *I*, *gnrr-2*(*tm4867*) *V*, and *gnrr-3*(*tm4152*) *X* were obtained from the National Bioresource Project for the Nematode courtesy of Dr. S. Mitani at Tokyo Women's Medical University School of Medicine. Strains containing *gnrr-1*(*ok238*) *I*, *ntr-1*(*ok2780*) *I*, *egl-15*(*n484*) *X*, *ver-1*(*ok1738*) *III*, *ver-2*(*ok897*) *I*, *ver-3*(*ok891*) *X*, and *ver-4*(*ok1079*) *X* were obtained from the *C. elegans* Genetics Center, which is funded by the NIH National Center for Research Resources (NCRR). N2 (Bristol) strain was utilized as a reference wild-type strain. Strains were outcrossed 4 times to the wild-type background. Unless described otherwise, strains were cultivated on NGM-agar plates seeded with *E. coli* OP-50 as described [63].

### RNA Interference

Synchronized first larval stage *C. elegans* were cultured for 3 d at 20°C on a lawn of HT115 *E. coli* induced with IPTG to express double-stranded RNAi as described [64].

### Pharyngeal Pumping Assay

Compounds at 1,000× stock concentrations (0.1–10 mM) in DMSO or an equal volume of DMSO were diluted in a suspension of *E. coli* OP-50 (100 µl of a 3× concentrated overnight culture in LB broth) and absorbed as a single drop onto 3.5 cm plates containing 2.5 ml of NGM agar forming a well-defined lawn of bacteria. For developmental exposures, 20–30 synchronized first larval stage *C. elegans* derived from alkaline hypochlorite treatment of gravid adults were applied to the preseeded plates and cultured until assay at mid-L4 stage (2 d, 20°C) or as day 1 gravid adults (3 d, 20°C). For naïve adult exposures, synchronized first larval stage animals were cultured on NGM-agar plates seeded with *E. coli* OP-50 for 3 d at 20°C, then transferred to assay plates, and pumping was assayed 1 to 16 h later. Comparisons between vehicle-treated and compound-treated animals were always performed between animals at the same developmental stage and same exposure time. Pharyngeal pumping was counted by live observation at 115–200× magnification using a stereo microscope and recorded at 10 s intervals using a manually controlled digital cell counter. Alternatively, *C. elegans* pumping was recorded for longer intervals (30–60 s) using bright-field time-lapse microscopy at 120× magnification with a 20 images per second acquisition rate. The resulting movies were analyzed manually at a playback rate of 10 images per second.

### Statistics

Significance was determined by one-way ANOVA applying a Dunnett's posttest when comparing multiple treatments to a single control and a Bonferroni posttest when comparing the more than two treatments against one another. For pairwise nonrepeated measured comparisons, a student's *t* test was used. To express data as a percentage of control, the pumping rates of compound-treated animals were divided by the mean pumping rate of the DMSO-treated wild-type animals measured in the same experiment, unless indicated otherwise. Error bars on the control samples indicate the variation around that mean, which is utilized in all statistical calculations.

## Supporting Information

Figure S1 **Dose-response measurements that validate SEA predictions for GPCRs.** (A) B16 inhibits rat mGluR-8 activity in CHO cells that were stimulated with 1 µM L-AP4 agonist. Error bars represent the s.e.m. of eight measurements. One-way ANOVA (Bonferonni) at concentrations of B16>3×10^−6^ M indicate the responses are significant (*p*<0.001). (B) Inhibition of ^3^H-oxytocin binding to the human oxytocin receptor by unlabeled oxytocin peptide (black squares) or F15 (red triangles). (C) H6 inhibits the calcium flux induced in cells expressing the human tachykinin-1 receptor, stimulated by neurokinin-1 peptide. Error bars represent the s.d. of three measurements. One-way ANOVA (Bonferroni) at concentrations >10^−6.4^ M indicate the responses are significant (*p*<0.01). (D) Inhibition of ^3^H-risperidone binding to the human dopamine D4 receptor by chlorpromazine (black squares) or G7 (red triangles) at concentrations of G7>10^−6.4^ M indicates the response is significant: *p*<0.001 ANOVA (Bonferroni).
(TIF)

Figure S2 **Dose-dependent inhibitory activity for compounds predicted by SEA to inhibit kinases.** (A–B) Varying concentrations of J6 and L10 were incubated with full-length recombinant human PI3KCA and ATP (50 µM) for 5 h and the phosphorylation level of the substrate phosphatidylinositol was measured. (A) L10 inhibition of PI3-kinase p110α is significant at concentrations >1×10^−6^ M (*p*<0.05, *t* test). (B) J6 inhibition of PI3-kinase p110α is significant at concentrations >1×10^−6^ M (*p*<0.05, *t* test). (C) D20 inhibition of flt-3 receptor catalytic domain activity. Varying concentrations of D20 were incubated with recombinant human Flt-3 receptor catalytic domain (amino acids 564–993), 100 µM ATP, and the phosphorylation of srctide peptide substrate was measured after 1 h at each concentration of D20. Inhibition at concentrations >1×10^−7^ M is significant (*p*<0.01, *t* test). Error bars represent the s.d. of two replicates.
(TIF)

Figure S3 **Dose-response relationships for compounds that bind nuclear hormone receptors.** (A) ^3^H-Rosiglitazone binding to human PPAR-γ in the presence of varying concentrations of I10. (B) ^3^H-Mibolerone binding to the human androgen receptor in the presence of varying concentrations of G6. Error bars represent the standard deviation of two replicates.
(TIF)

Figure S4 **Measurement of the pharyngeal pumping effects of the compounds by time lapse microscopy.** *C. elegans* were cultured for 3 d from L1 larvae in the presence of either 10 µM B16, H6, D20, and F15 or 0.1% DMSO as the vehicle control. Pharyngeal pumping over 60 s intervals was measured in time lapse recordings of at least 60 s in duration. A comparison with the pumping rates measured over 10 s (6-fold extrapolated) by real-time direct observation of the same populations of animals is presented. Real-time, 10 s interval manual counting involves a systematic underestimation of pumping across all conditions, however the relative ratios are similar. Ten animals were measured per condition. Error bars represent the standard deviation. *p<0.001 one-way ANOVA, Bonferroni posttest.
(TIF)

Figure S5 **Effect on pharyngeal pumping of combinations of compounds that target the same human receptor.** (A) *C. elegans* were cultured with DMSO (0.2%), 10 µM D20, 1 µM 5-flurox, or a combination of 10 µM D20 and 1 µM 5-flurox. (B) *C. elegans* were cultured with DMSO (0.2%), 10 µM F15, 200 nM L-371257, or a combination of 10 µM F15 and 200 nM L-371257. (C) *C. elegans* were cultured with DMSO (0.2%), 10 µM H6, 200 nM SB222200, or a combination of 10 µM H6 and 200 nM SB222200. (D) *C. elegans* were cultured with DMSO (0.2%), 10 µM B16, 2 µM MMPIP, or a combination of 10 µM H6 and 2 µM MMPIP. In (A–D) 12 animals per condition were counted. Error bars represent the standard deviation. *p<0.001 ANOVA, Dunnett's posttest.
(TIF)

Figure S6 **Genetic interactions of** ***gnrr-1*** **mutants quantified by time lapse measurements.** Wild-type and *gnrr-1* mutants were cultured on bacteria expressing double-stranded RNA targeting *mgl-2*, *ver-3*, *ver-2*, or the RNAi expression vector control. Pharyngeal pumping of 10 animals per condition for 30 s intervals was recorded by time-lapse microscopy. The color scheme of the figure matches that of Figure 5. Error bars represent the standard deviation. ns, not specific, *p*>0.05, *p<0.05, **p<0.01 ANOVA, Bonferroni posttest.
(TIF)

Movie S1 ***C. elegans*** **treated with 10 µM F15.**
(MP4)

Movie S2 ***C. elegans*** **treated with 10 µM B16.**
(MP4)

Movie S3 ***C. elegans*** **treated with 10 µM D20.**
(MP4)

Movie S4 ***C. elegans*** **treated with 10 µM H6.**
(MP4)

Movie S5 ***C. elegans*** **treated with 0.1% DMSO.**
(MP4)

Table S1 **Target predictions that could not be confirmed** ***in vitro*** **.**
(TIF)

Table S2 **Activity of 10 µM B16 on human metabotropic glutamate receptors.**
(DOCX)

Table S3 **Known expression patterns of** ***C. elegans*** **homologs used in the manuscript and their sequence comparisons to human targets.**
(DOCX)

Table S4 **Epistasis data used to construct interaction matrix in** **Figure 6A** **.**
(DOC)

## Abbreviations

5-flurox: 5′-fluoroindirubinoxime
ANOVA: analysis of variance
BLAST: Basic Local Alignment Search Tool
ECFP: extended-connectivity fingerprint
Flt-3: Fms-like tyrosine kinase 3
GPCR: G-protein coupled receptor
L1: first larval stage
mGluR: metabotropic glutamate receptor
NHR: nuclear hormone receptor
Oxtr: oxytocin receptor
PDGF-β: platelet-derived growth factor-β
PI3K: phosphoinositide 3-kinase
PPAR: peroxisome proliferator activator receptor
SEA: Similarity Ensemble Approach
Tkr: tachykinin receptor
VEGF: vascular endothelial growth factor
