# Supplementary figures and images for: In Silico Molecular Comparisons of C. elegans and Mammalian Pharmacology Identify Distinct Targets That Regulate Feeding
Source: PLoS Biol. 2013 Nov 19;11(11):e1001712. doi: 10.1371/journal.pbio.1001712 (PMC3833878; doi:10.1371/journal.pbio.1001712)

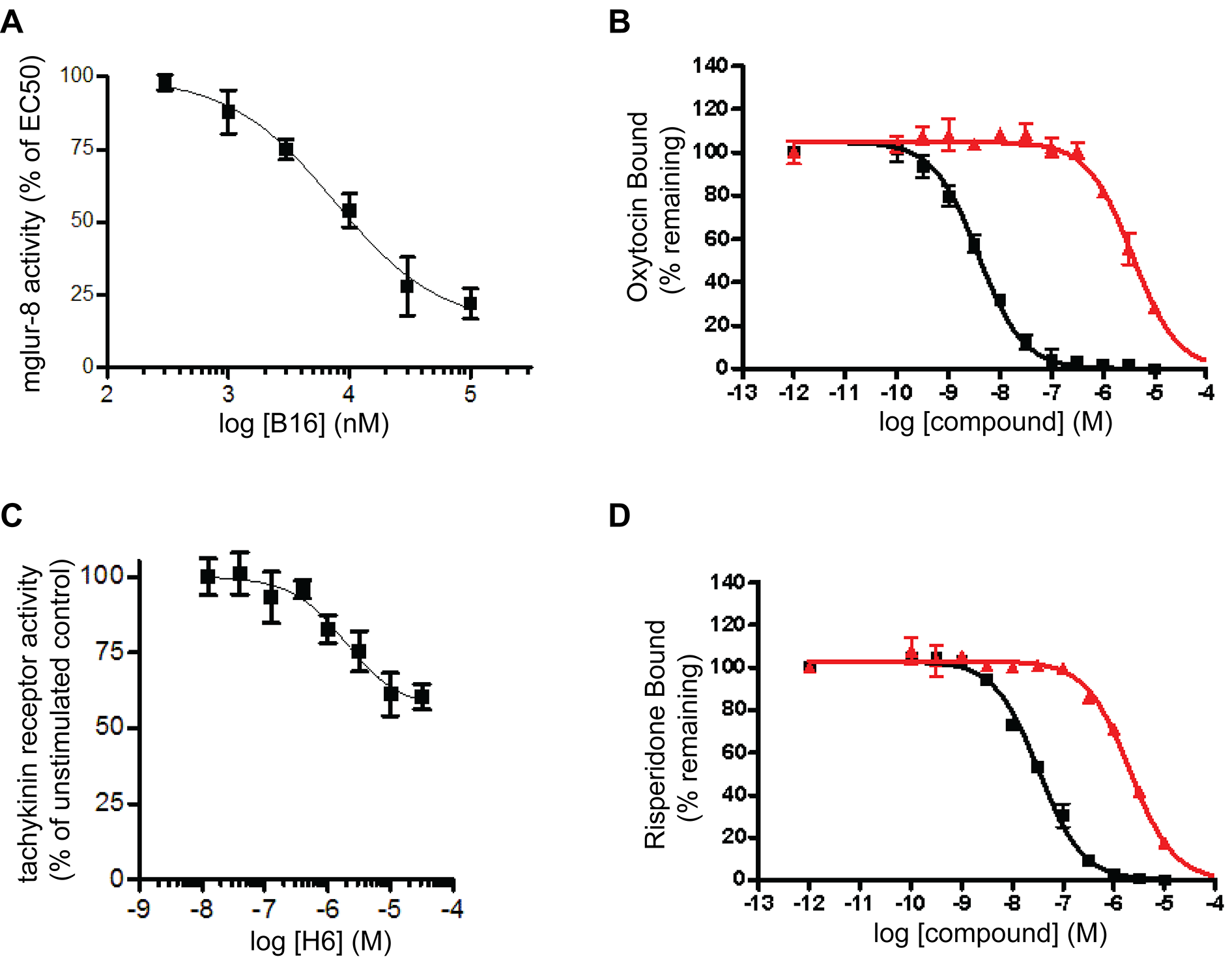

Supplement: Figure S1 — Dose-response measurements that validate SEA predictions for GPCRs. (A) B16 inhibits rat mGluR-8 activity in CHO cells that were stimulated with 1 µM L-AP4 agonist. Error bars represent the s.e.m. of eight measurements. One-way ANOVA (Bonferonni) at concentrations of B16>3×10^−6^ M indicate the responses are significant (p<0.001). (B) Inhibition of 3H-oxytocin binding to the human oxytocin receptor by unlabeled oxytocin peptide (black squares) or F15 (red triangles). (C) H6 inhibits the calcium flux induced in cells expressing the human tachykinin-1 receptor, stimulated by neurokinin-1 peptide. Error bars represent the s.d. of three measurements. One-way ANOVA (Bonferroni) at concentrations >10−6.4 M indicate the responses are significant (p<0.01). (D) Inhibition of 3H-risperidone binding to the human dopamine D4 receptor by chlorpromazine (black squares) or G7 (red triangles) at concentrations of G7>10−6.4 M indicates the response is significant: p<0.001 ANOVA (Bonferroni). (TIF) [file pbio.1001712.s001.tif]

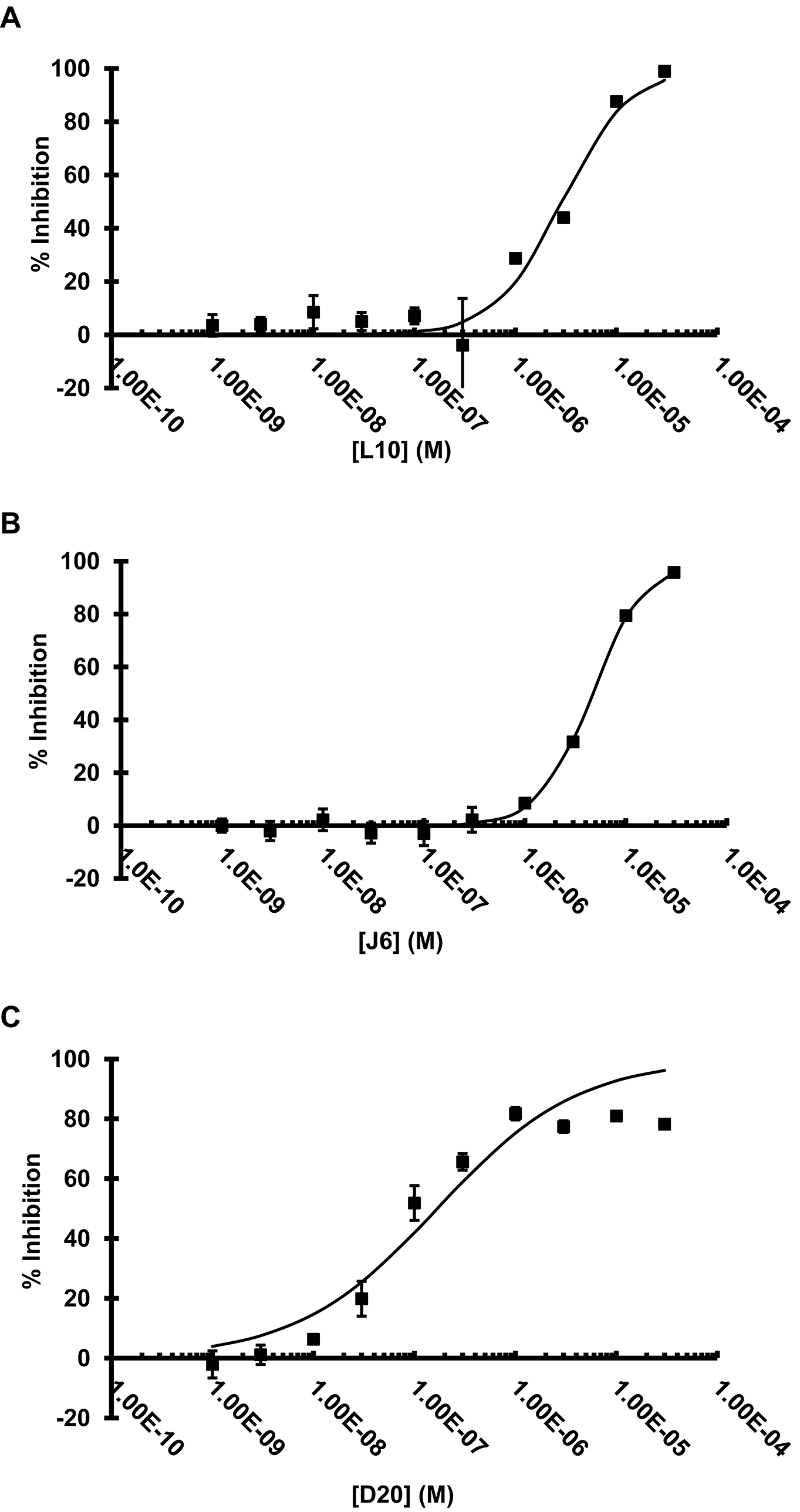

Supplement: Figure S2 — Dose-dependent inhibitory activity for compounds predicted by SEA to inhibit kinases. (A–B) Varying concentrations of J6 and L10 were incubated with full-length recombinant human PI3KCA and ATP (50 µM) for 5 h and the phosphorylation level of the substrate phosphatidylinositol was measured. (A) L10 inhibition of PI3-kinase p110α is significant at concentrations >1×10^−6^ M (p<0.05, t test). (B) J6 inhibition of PI3-kinase p110α is significant at concentrations >1×10^−6^ M (p<0.05, t test). (C) D20 inhibition of flt-3 receptor catalytic domain activity. Varying concentrations of D20 were incubated with recombinant human Flt-3 receptor catalytic domain (amino acids 564–993), 100 µM ATP, and the phosphorylation of srctide peptide substrate was measured after 1 h at each concentration of D20. Inhibition at concentrations >1×10^−7^ M is significant (p<0.01, t test). Error bars represent the s.d. of two replicates. (TIF) [file pbio.1001712.s002.tif]

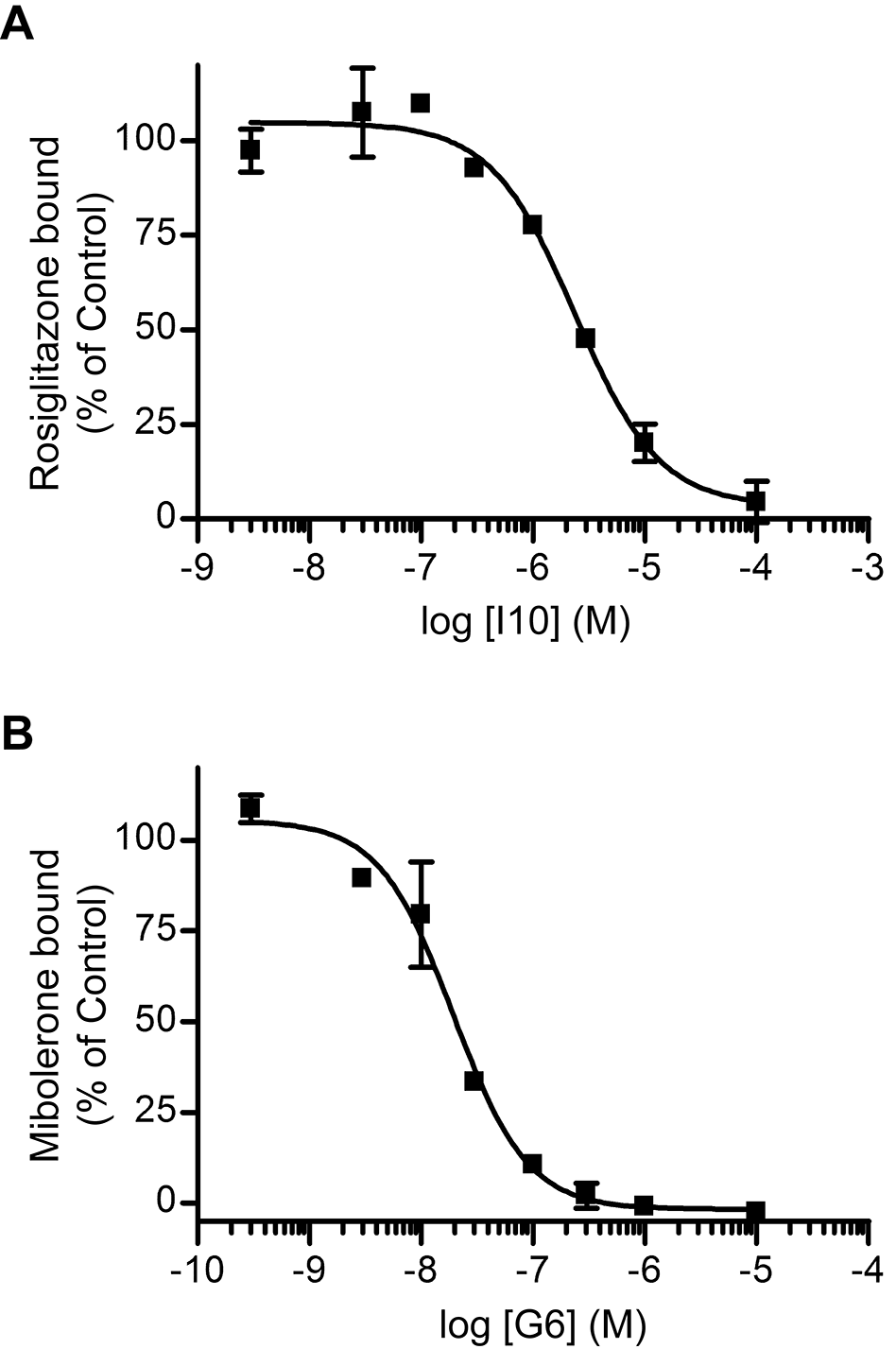

Supplement: Figure S3 — Dose-response relationships for compounds that bind nuclear hormone receptors. (A) 3H-Rosiglitazone binding to human PPAR-γ in the presence of varying concentrations of I10. (B) 3H-Mibolerone binding to the human androgen receptor in the presence of varying concentrations of G6. Error bars represent the standard deviation of two replicates. (TIF) [file pbio.1001712.s003.tif]

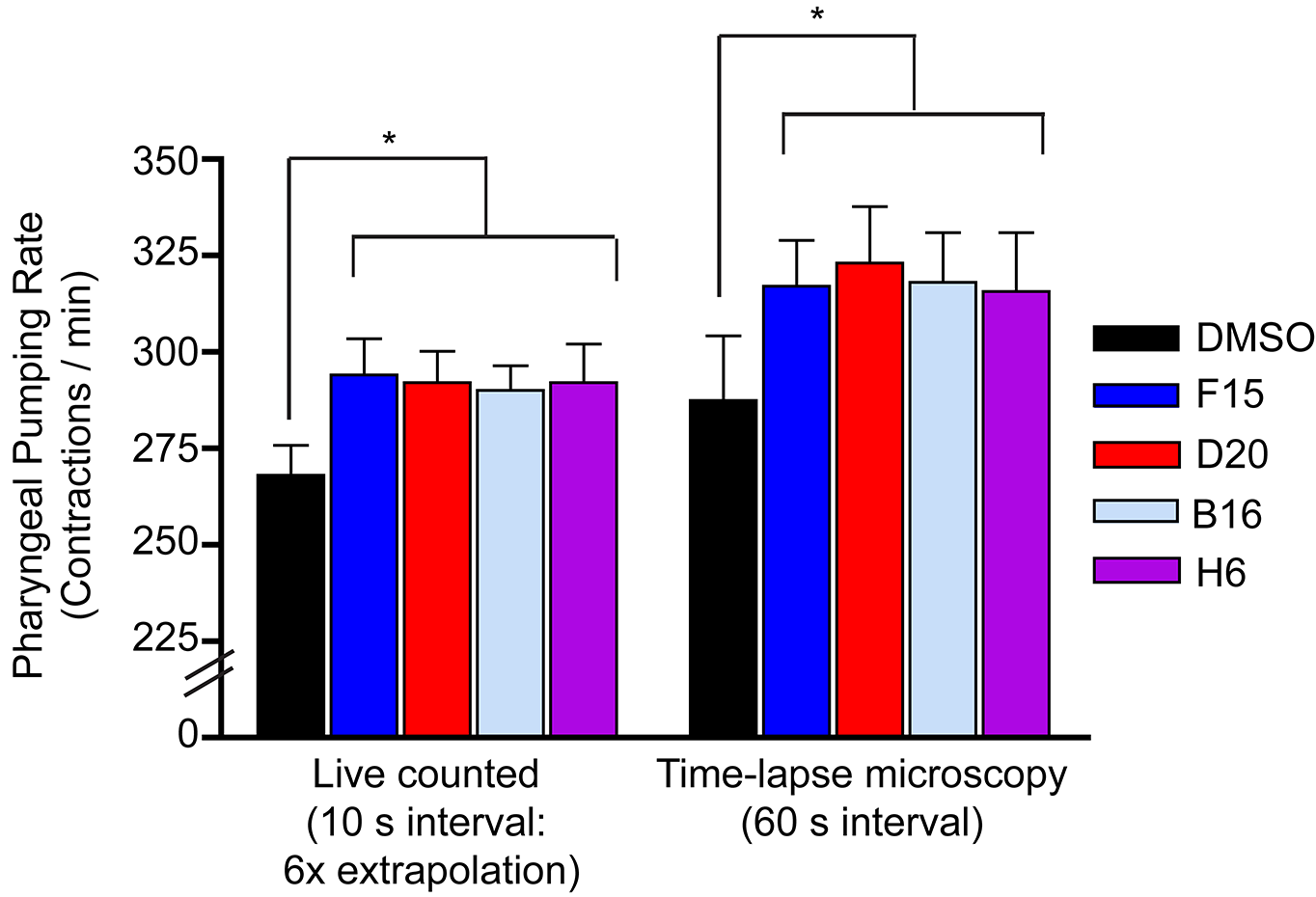

Supplement: Figure S4 — Measurement of the pharyngeal pumping effects of the compounds by time lapse microscopy. C. elegans were cultured for 3 d from L1 larvae in the presence of either 10 µM B16, H6, D20, and F15 or 0.1% DMSO as the vehicle control. Pharyngeal pumping over 60 s intervals was measured in time lapse recordings of at least 60 s in duration. A comparison with the pumping rates measured over 10 s (6-fold extrapolated) by real-time direct observation of the same populations of animals is presented. Real-time, 10 s interval manual counting involves a systematic underestimation of pumping across all conditions, however the relative ratios are similar. Ten animals were measured per condition. Error bars represent the standard deviation. *p<0.001 one-way ANOVA, Bonferroni posttest. (TIF) [file pbio.1001712.s004.tif]

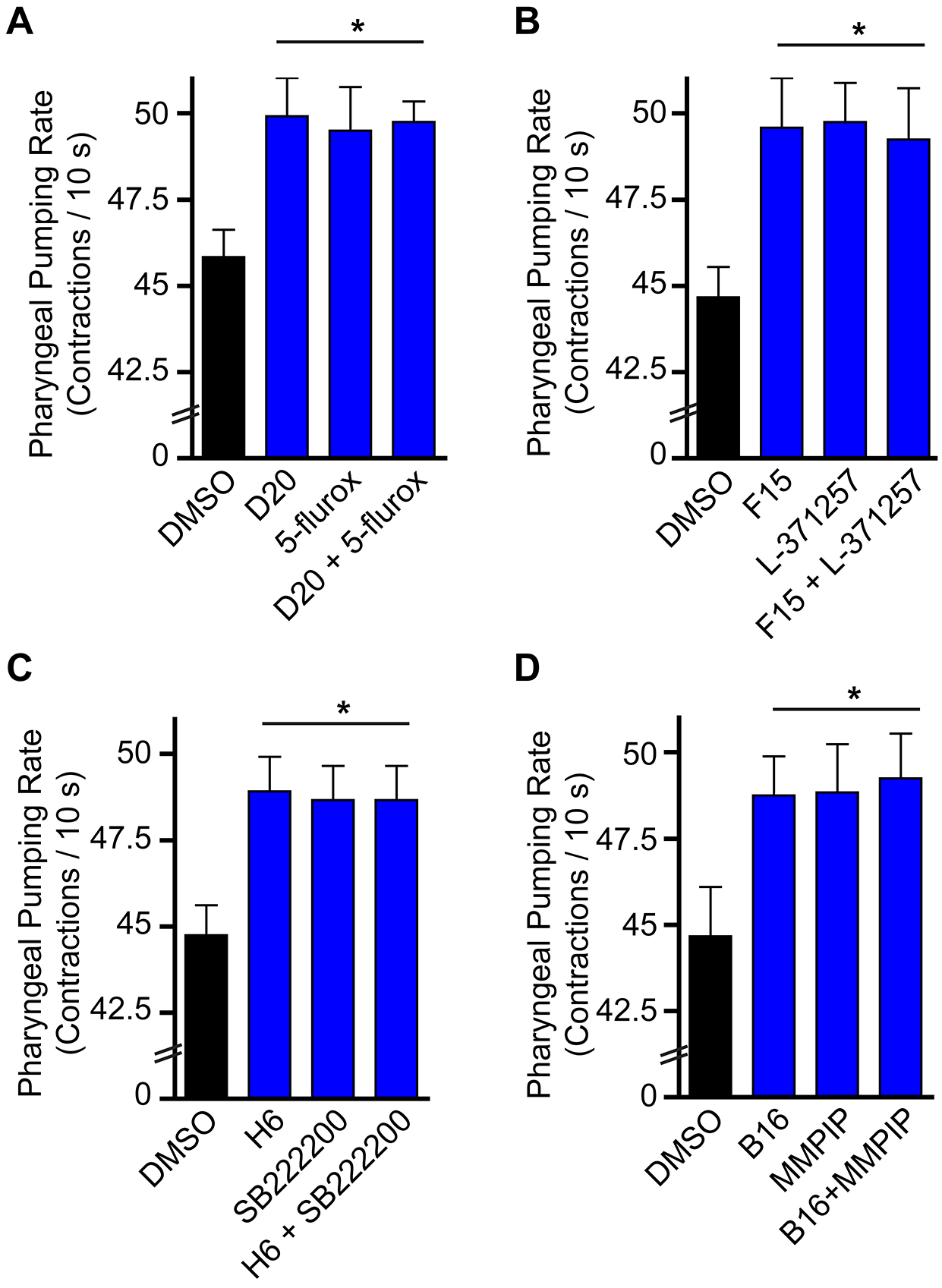

Supplement: Figure S5 — Effect on pharyngeal pumping of combinations of compounds that target the same human receptor. (A) C. elegans were cultured with DMSO (0.2%), 10 µM D20, 1 µM 5-flurox, or a combination of 10 µM D20 and 1 µM 5-flurox. (B) C. elegans were cultured with DMSO (0.2%), 10 µM F15, 200 nM L-371257, or a combination of 10 µM F15 and 200 nM L-371257. (C) C. elegans were cultured with DMSO (0.2%), 10 µM H6, 200 nM SB222200, or a combination of 10 µM H6 and 200 nM SB222200. (D) C. elegans were cultured with DMSO (0.2%), 10 µM B16, 2 µM MMPIP, or a combination of 10 µM H6 and 2 µM MMPIP. In (A–D) 12 animals per condition were counted. Error bars represent the standard deviation. *p<0.001 ANOVA, Dunnett's posttest. (TIF) [file pbio.1001712.s005.tif]

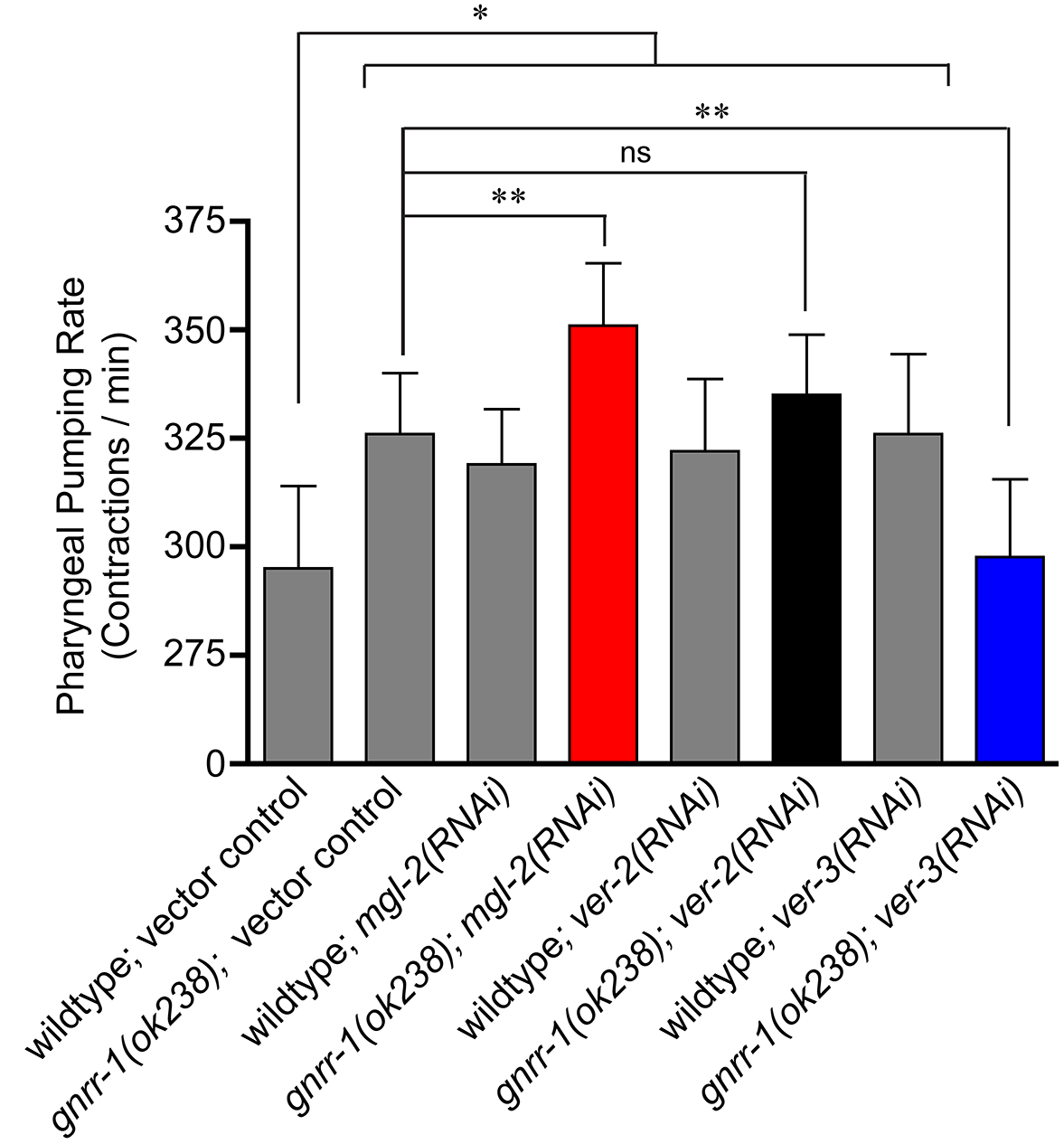

Supplement: Figure S6 — Genetic interactions of gnrr-1 mutants quantified by time lapse measurements. Wild-type and gnrr-1 mutants were cultured on bacteria expressing double-stranded RNA targeting mgl-2, ver-3, ver-2, or the RNAi expression vector control. Pharyngeal pumping of 10 animals per condition for 30 s intervals was recorded by time-lapse microscopy. The color scheme of the figure matches that of Figure 5. Error bars represent the standard deviation. ns, not specific, p>0.05, *p<0.05, **p<0.01 ANOVA, Bonferroni posttest. (TIF) [file pbio.1001712.s006.tif]

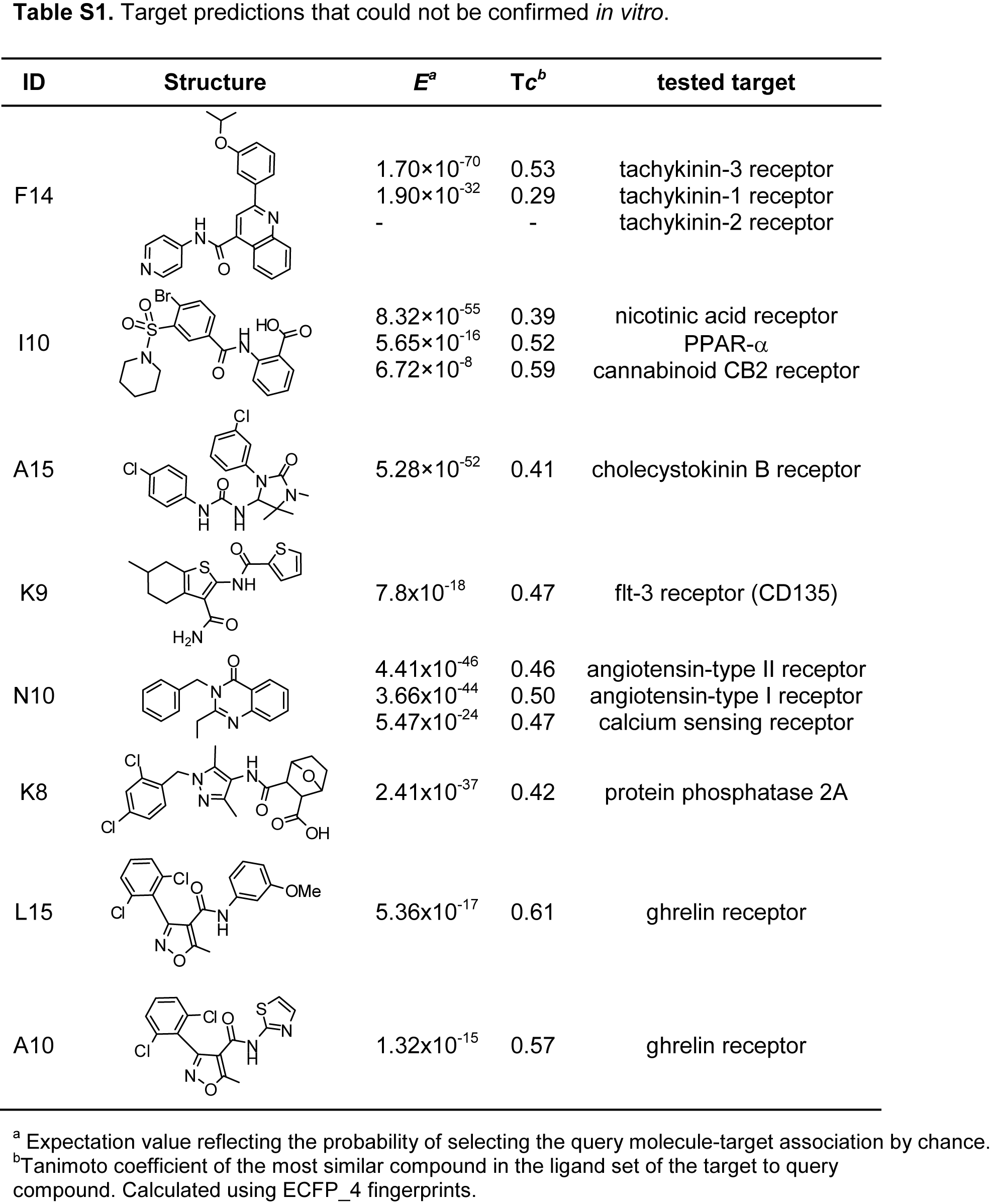

Supplement: Table S1 — Target predictions that could not be confirmed in vitro . (TIF) [file pbio.1001712.s012.tif]
