# Supplementary material for: In Silico Molecular Comparisons of C. elegans and Mammalian Pharmacology Identify Distinct Targets That Regulate Feeding
Source: PLoS Biol. 2013 Nov 19;11(11):e1001712. doi: 10.1371/journal.pbio.1001712 (PMC3833878; doi:10.1371/journal.pbio.1001712)
Supplement: Table S2 — Activity of 10 µM B16 on human metabotropic glutamate receptors. (DOCX) [file pbio.1001712.s013.docx]

| **receptor** | **% agonism^a^** | **% inhibition^b^** |
| --- | --- | --- |
| MGLUR1a | -4 | 24 |
| MGLUR2 | 0 | 12 |
| MGLUR4 | -8 | 4 |
| MGLUR5 | -14 | 11 |
| MGLUR6 | 13 | -17 |
| MGLUR8 | -3 | 49 |

**Table S2**. Activity of 10 μM B16 on human metabotropic glutamate receptors.

^a^ Data are normalized to the maximum response of cells treated with 1 mM glutamate or 0.1 mM AP4 alone.

^b^ Percent difference in maximum activity of cells treated with glutamate or AP4 with B16 vehicle versus concurrent treatment with 10 μM B16.
