# Supplementary material for: In Silico Molecular Comparisons of C. elegans and Mammalian Pharmacology Identify Distinct Targets That Regulate Feeding
Source: PLoS Biol. 2013 Nov 19;11(11):e1001712. doi: 10.1371/journal.pbio.1001712 (PMC3833878; doi:10.1371/journal.pbio.1001712)
Supplement: Table S3 — Known expression patterns of C. elegans homologs used in the manuscript and their sequence comparisons to human targets. (DOCX) [file pbio.1001712.s014.docx]

**Table S3.** Sequence relationships and reported expression patterns between feeding increasing compounds and the *C. elegans* genes examined in this study.

|  | **Nile Red staining** | **human target** | ***E* (BLASTp)** | **% identity** | **Cosmid #** | **name** | **Pharmacological relationship** | ***C. elegans*' expression pattern^a^** |
| --- | --- | --- | --- | --- | --- | --- | --- | --- |
| **B16** | decreased | mGluR-8 | 0 | 37 | ZC506.4 | *mgl-1* |  | interneurons, motor neurons, pharyngeal neurons |
|  |  |  | 0 | 38 | Y4C6A.2 | *mgl-3* |  | neurons |
|  |  |  | 1 x10^-167^ | 35 | F45H11.4 | *mgl-2* | antagonist | interneurons |
| **H6** | increased | Tkr-1 | 7 x10^-57^ | 33 | C38C10.1 | *tkr-1* | antagonist | deirid sheath cells |
| **F15** | decreased | Oxtr | 5 x10^-31^ | 28 | T07D10.2 | *ntr-1* |  | ADF, ADL, RIC head neurons, male: HOB neurons and male tail rays^c^ ASEL, ADF, ASH, PQR^d^ |
|  |  |  | 3 x10^-30^ | 29 | F54D7.3 | *gnrr-1* | antagonist | head neurons, anterior pharynx, ventral nerve cord pharyngeal muscle, oocyte nuclei, intestinal nuclei, sperm |
|  |  |  | 2 x10^-17^ | 23 | C38C10.1 | *tkr-1* |  | deirid sheath cells |
|  |  |  | 9 x10^-15^ | 23 | C15H11.2 | *gnrr-2* |  | unknown |
|  |  |  | 4 x10^-21b^ | 24^b^ | ZC374.1 | *gnrr-3* |  | unknown |
| **D20** | decreased | Flt-3 receptor | 5 x10^-67^ | 37 | F58A3.2 | *egl-15* |  | hypodermis, intestine, vulval muscle, neurons |
|  |  |  | 1 x10^-57^ | 34 | C24G6.2 | *-* |  | unknown |
|  |  |  | 1 x10^-50^ | 32 | M176.7 | *kin-16* |  | hypodermis, intestine |
|  |  |  | 1 x10^-48^ | 34 | M176.6 | *kin-15* |  | hypodermis, intestine |
|  |  |  | 5 x10^-47^ | 33 | F59F3.1 | *ver-3* | antagonist | ALA neuron, pharyngeal saucer muscle, and anal sphincter muscle |
|  |  |  | 2 x10^-45^ | 31 | F08F1.1 | *kin-9* |  | unknown |
|  |  |  | 3 x10^-46^ | 28 | F59F3.5 | *ver-4* |  | unknown |
|  |  |  | 9 x10^-45^ | 32 | M01B2.1 | *kin-30* |  | unknown |
|  |  |  | 2 x10^-43^ | 32 | ZK938.5 | *old-2* |  | unknown |
|  |  |  | 6 x10^-43^ | 34 | W04G5.6 | *kin-23* |  | unknown |
|  |  |  | 6 x10^-43^ | 32 | F09A5.2 | *-* |  | uknown |
|  |  |  | 7 x10^-41^ | 27 | T17A3.1 | *ver-1* |  | amphid and phasmid sheath cells |
|  |  |  | 3 x10^-38^ | 45 | M79.1 | *abl-1* |  | Pharynx, ventral nerve cord, germ cells |
|  |  |  | 4 x10^-38^ | 43 | C08H9.5 | *old-1* |  | Hypodermis, neurons, pharynx, male gonad |
|  |  |  | 7 x10^-36^ | 47 | F49B2.5 | *src-2* |  | Pharyngeal muscles, vulva, anus |
|  |  |  | 2 x10^-33^ | 41 | F11E6.8 | *-* |  | unknown |
|  |  |  | 3 x10^-32^ | 28 | T17A3.8 | *ver-2* |  | ADL neuron |
|  |  |  | 1 x10^-30^ | 44 | ZK622.1 | *-* |  | unknown |
|  |  |  | 2 x10^-30^ | 44 | F09G2.1 | *-* |  | unknown |
|  |  |  | 2 x10^-30^ | 25 | C01G6.8 | *cam-1* |  | Multiple head neurons, muscles |
|  |  |  | 3 x10^-29^ | 37 | ZK1067.1 | *let-23* |  | ALA, head neuron, tail neuron, intestinal valve, posterior arcade cell |
|  |  |  | 7 x10^-29^ | 34 | T04B2.2 | *frk-1* |  | Body wall muscle, sperm, germline, epithelial cell |
|  |  |  | 8 x10^-27^ | 39 | F22D6.1 | *kin-14* |  | unknown |
|  |  |  | 8 x10^-25^ | 36 | M03A1.1 | *vab-1* |  | Head neurons, ventral nerve cord, oocytes & gonad sheath cells |
|  |  |  | 5 x10^-24^ | 40 | F57B9.8 | *-* |  | unknown |
|  |  |  | 6 x10^-24^ | 40 | F22D6.1 | *ddr-2* |  | Few head neurons, seam cells, hypodermis |

^a^As described in WormBase (<http://www.wormbase.org>)

^b^relative to F54D7.3 (GNRR-1) query

^c^from Garrison, J.L. *et al.* (2012) *Science* **338**, p540-543

^d^from Beets, I. *et al.* (2012) *Science* **338**, p543-545
