# Supplementary material for: In Silico Molecular Comparisons of C. elegans and Mammalian Pharmacology Identify Distinct Targets That Regulate Feeding
Source: PLoS Biol. 2013 Nov 19;11(11):e1001712. doi: 10.1371/journal.pbio.1001712 (PMC3833878; doi:10.1371/journal.pbio.1001712)
Supplement: Table S4 — Epistasis data used to construct interaction matrix in Figure 6A . (DOC) [file pbio.1001712.s015.doc]

**Table S4.** Epistasis data used to construct the interaction matrix in Figure 6A.

|  | *mgl-2(tm355)* | | | | *ver-3(ok891)* | | | | *gnrr-1(ok238)* | | | | *ver-2(ok897)* | | | | *tkr-1(RNAi)* | | | | *gnrr-3(tm4152)* | | | |
| --- | --- | --- | --- | --- | --- | --- | --- | --- | --- | --- | --- | --- | --- | --- | --- | --- | --- | --- | --- | --- | --- | --- | --- | --- |
|  | mean | stdev | n | p | mean | stdev | n | p | mean | stdev | n | p | mean | stdev | n | p | mean | stdev | n | p | mean | stdev | n | p |
| DMSO | 100.0 | 2.8 | 16 |  | 100.0 | 2.3 | 16 |  | 100.0 | 2.6 | 32 |  | 100.0 | 3.4 | 16 |  | 100.0 | 1.5 | 21 |  | 100.0 | 3.2 | 16 |  |
| B16 | 100.0 | 3.0 | 12 | > 0.05 | 99.5 | 2.2 | 14 | >0.05 | 105.0 | 2.7 | 16 | <0.05 | 90.5 | 7.9 | 16 | <0.05 | 105.1 | 2.1 | 21 | >0.05 | 106.2 | 2.3 | 16 | >0.05 |
| MMPIP | 99.8 | 2.1 | 12 | >0.05 | 99.7 | 5.3 | 16 | >0.05 | 105.0 | 3.2 | 16 | <0.05 | 92.5 | 3.2 | 16 | <0.05 | 107.2 | 3.7 | 16 | >0.05 | 106.9 | 3.6 | 16 | >0.05 |
| D20 | 99.0 | 1.8 | 16 | >0.05 | 99.6 | 3.1 | 23 | >0.05 | 93.5 | 3.9 | 16 | <0.05 | 93.1 | 3.0 | 14 | <0.05 | 107.8 | 3.1 | 14 | >0.05 | 109.1 | 2.9 | 16 | >0.05 |
| K9 | 99.3 | 2.9 | 16 | >0.05 | 100.2 | 3.2 | 22 | >0.05 | 94.5 | 2.0 | 16 | <0.05 | 92.0 | 2.2 | 12 | <0.05 | 109.6 | 3.3 | 13 | >0.05 | 107.8 | 2.7 | 12 | >0.05 |
| 5-flurox | 100.0 | 3.9 | 16 | >0.05 | 100.5 | 3.2 | 21 | >0.05 | 92.5 | 2.4 | 16 | <0.05 | 91.4 | 4.8 | 14 | <0.05 | 107.8 | 4.2 | 14 | >0.05 | 109.2 | 1.6 | 12 | >0.05 |
| F15 | 106.3 | 3.9 | 12 | <0.05 | 95.2 | 3.8 | 16 | <0.05 | 100.4 | 3.8 | 21 | >0.05 | 99.3 | 4.0 | 16 | >0.05 | 107.6 | 3.7 | 15 | >0.05 | 107.8 | 3.0 | 16 | >0.05 |
| L-371257 | 105.0 | 3.0 | 14 | <0.05 | 94.8 | 3.1 | 16 | <0.05 | 99.2 | 3.4 | 12 | >0.05 | 100.8 | 3.2 | 16 | >0.05 | 109.9 | 3.1 | 16 | >0.05 | 108.5 | 4.1 | 16 | >0.05 |
| H6 | 105.3 | 2.5 | 16 | <0.05 | 105.9 | 3.6 | 16 | <0.05 | 108.6 | 2.6 | 12 | <0.05 | 104.2 | 4.2 | 16 | >0.05 | 100.3 | 2.2 | 18 | <0.05 | 105.8 | 3.3 | 16 | >0.05 |
| SB222200 | 105.8 | 3.3 | 16 | <0.05 | 107.1 | 1.4 | 10 | <0.05 | 108.6 | 3.8 | 12 | <0.05 | 105.7 | 3.8 | 26 | >0.05 | 100.5 | 1.5 | 17 | <0.05 | 108.5 | 2.5 | 16 | >0.05 |

Each panel displays data quantifying the effect on pharyngeal pumping of each feeding increasing compound used in this study on a given mutant which has been identified as high-feeding relative to wild-type. The pumping rates are expressed as a percentage of the mutant’s pumping rate on DMSO, the vehicle control. p-values were calculated relative to DMSO treatment of each mutant by one-way ANOVA using Dunnett’s multiple comparison test.
